# Fast detection and structural identification of carbocations on zeolites by dynamic nuclear polarization enhanced solid-state NMR†
†Electronic supplementary information (ESI) available. See DOI: 10.1039/c8sc03848a


**DOI:** 10.1039/c8sc03848a

**Published:** 2018-10-02

**Authors:** Dong Xiao, Shutao Xu, Nick J. Brownbill, Subhradip Paul, Li-Hua Chen, Shane Pawsey, Fabien Aussenac, Bao-Lian Su, Xiuwen Han, Xinhe Bao, Zhongmin Liu, Frédéric Blanc

**Affiliations:** a State Key Laboratory of Catalysis , Dalian Institute of Chemical Physics , Chinese Academy of Sciences , 457 Zhongshan Road , Dalian 116023 , China; b University of Chinese Academy of Sciences , Beijing 100049 , China; c Department of Chemistry , University of Liverpool , Crown Street , Liverpool , L69 7ZD , UK . Email: frederic.blanc@liverpool.ac.uk; d National Engineering Laboratory for Methanol to Olefins , Dalian National Laboratory for Clean Energy , Dalian Institute of Chemical Physics , Chinese Academy of Sciences , Dalian 116023 , China; e DNP MAS NMR Facility , Sir Peter Mansfield Imaging Centre , University of Nottingham , Nottingham NG7 2RD , UK; f State Key Laboratory of Advanced Technology for Materials Synthesis and Processing , Wuhan University of Technology , 122 Luoshi Road , 430070 , Wuhan , China; g Bruker BioSpin Corporation , 15 Fortune Drive , Billerica , Massachusetts 01821 , USA; h Bruker BioSpin , 34 rue de I'Industrie BP 10002 , 67166 Wissembourg Cedex , France; i CMI (Laboratory of Inorganic Materials Chemistry) , University of Namur , 61 rue de Bruxelles , B-5000 Namur , Belgium; j Stephenson Institute for Renewable Energy , University of Liverpool , Crown Street , Liverpool L69 7ZD , UK

## Abstract

Acidic zeolites are porous aluminosilicates used in a wide range of industrial processes such as adsorption and catalysis. The formation of carbocation intermediates plays a key role in reactivity, selectivity and deactivation in heterogeneous catalytic processes. However, the observation and determination of carbocations remain a significant challenge in heterogeneous catalysis due to the lack of selective techniques of sufficient sensitivity to detect their low concentrations. Here, we combine ^13^C isotopic enrichment and efficient dynamic nuclear polarization magic angle spinning nuclear magnetic resonance spectroscopy to detect carbocations in zeolites. We use two dimensional ^13^C–^13^C through-bond correlations to establish their structures and ^29^Si–^13^C through-space experiments to quantitatively probe the interaction between multiple surface sites of the zeolites and the confined hydrocarbon pool species. We show that a range of various membered ring carbocations are intermediates in the methanol to hydrocarbons reaction catalysed by different microstructural β-zeolites and highlight that different reaction routes for the formation of both targeted hydrocarbon products and coke exist. These species have strong van der Waals interaction with the zeolite framework demonstrating that their accumulation in the channels of the zeolites leads to deactivation. These results enable understanding of deactivation pathways and open up opportunities for the design of catalysts with improved performances.

## Abstract 2

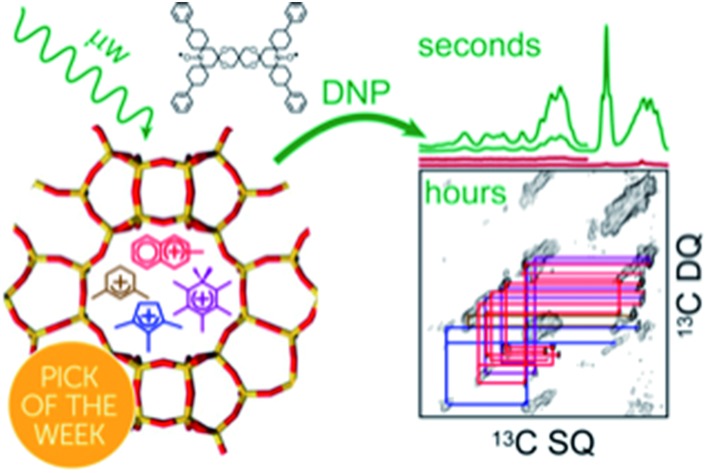
A fast NMR data acquisition strategy is explored to detect and characterize carbocations on solid zeolites.

## Introduction

Carbocations are important intermediates in many homogeneous^1^,^2^ and heterogeneous reactions,^3^–^6^ especially those catalysed by solid acids (*e.g.* acidic zeolites),^7^–^9^ and are formed from the corresponding hydrocarbons through protonation by the acidic protons of the Brønsted acid sites. They take part in a range of industrial processes such as cracking, isomerization, alkylation, *etc.*, which account for the conversion of hydrocarbons to a range of products.^8^ For example, cyclic carbocations are proposed as important intermediates involved in the hydrocarbon pool mechanism for the conversion of methanol to hydrocarbons (MTH).^9^–^17^ Despite the significant roles of carbocations in heterogeneous reactions, their identifications in solid catalysts are not straightforward as they are reactive, transient, difficult to capture and exist in generally low concentrations,^3^,^15^,^18^–^20^ and therefore their spectroscopic characterization is very challenging.^15^,^19^–^21^

Solid-state NMR is useful in detecting reactive carbocations on solid catalysts as shown in some limited cases on zeolites where their capture is achieved by quenching the reaction with liquid N_2_ (ref. 15 and 22) or stabilizing the intermediates with a base (*e.g.* ammonia).^20^ However, further development in extending the use of solid-state NMR to the study of carbocations is currently hindered by both the challenge associated with capturing enough highly reactive carbocations formed on solids (*vide supra*) and the intrinsically low sensitivity of NMR, especially when low natural abundance nuclei (*e.g.* 1.1% for ^13^C) are targeted. Although ^13^C isotopically enriched reagents are generally used to overcome this inherently poor sensitivity,^15^,^19^,^22^ the small amount of carbocations that can be captured in successful cases (typically 0.01 mmol g^–1^ in the MTH activated β-zeolite^19^) usually only permits the acquisition of one dimensional (1D) NMR signals, limiting the application of more informative multidimensional NMR experiments to obtain both the structures of these carbocations and their interaction with the solid catalysts. The structures of adsorbed carbocations are typically derived from such 1D ^13^C NMR spectra combined with gas chromatography-mass spectrometry (GC-MS) and density functional theory (DFT) calculations^15^,^16^ and therefore prior assumption of the existing structures is required.

We recently identified the carbocations formed in ^13^C enriched MTH activated ZSM-5 and investigated their host–guest interaction by obtaining limited structural constraints.^23^ However, the experimental times needed to acquire the multidimensional and multinuclear NMR data were prohibitively long (>5 days), even in this favourable case where the carbocation concentration is relatively high (>0.02 mmol g^–1^). This significantly hinders the systematic use of these powerful approaches on a wider range of solid acids with a lower amount of carbocation intermediates and addressing this challenge necessitates further dramatic boost in NMR sensitivity beyond ^13^C labelling.^21^

An emerging method with potential to delivering this increase in NMR sensitivity is dynamic nuclear polarization (DNP) which can enhance the NMR signals by multiple orders of magnitude by transferring the large polarization of electrons to nearby nuclei *via* microwave (μw) induced electron–nuclear transitions, thereby leading to a very significant reduction in experimental time.^24^–^30^ Insoluble samples for typical DNP experiments are impregnated with a solution of stable radicals as the source of electrons with the solvent providing the matrix for ^1^H polarization transfer. Although initial work focused on water-soluble radicals^29^,^30^ which are chemically incompatible with the investigated carbocations, water free radicals and matrices are now known^31^,^32^ and have enabled the structural characterization of a broad range of materials with DNP.^30^ In particular in heterogeneous catalysis, this approach is starting to provide detailed access to catalytic sites on the surface or in the pores of selected catalysts,^28^,^33^–^40^ including surface-enhanced NMR on mesoporous silica,^28^ organometallics on silica,^33^,^34^ Sn^VI^-active sites in the Sn-β zeolite^35^–^37^ and Brønsted acid sites of aluminosilicates.^38^ However, DNP investigation on reactive carbocation intermediates confined in microporous zeolites is yet to be demonstrated.

Here, we explore the use of DNP NMR to detect carbocations confined in porous zeolite catalysts during the MTH reaction.^16^,^19^ We show that combining ^13^C isotope enrichment and DNP enables the detection of low levels of carbocations (0.002–0.01 mmol g^–1^) in two types of β-zeolites within minutes. The considerable sensitivity increase obtained allows carbon connectivities to be obtained with ^13^C–^13^C through-bond experiments^41^ (see Fig. S1 in the ESI†) yielding the molecular structures of a series of carbocations. The spatial proximities between the surface sites of the zeolites and the confined carbon species are quantitatively probed *via*^13^C–^29^Si through-space NMR experiments.^42^ The identification of these carbocations reveals possible reaction routes for the formation of olefins and coke species in the MTH reaction while the quantification of host–guest interaction indicates dominant interaction contributing to the adsorption of hydrocarbon pool species in zeolites. In addition, while DNP application has been shown to be limited for microporous zeolites,^35^,^37^ here we suggest a potential way to optimize DNP efficiency on microporous zeolites, that is introducing hierarchical pores with different sizes ranging from micro (<2 nm), meso (2–50 nm) to macro pores (>50 nm).^43^

## Results and discussion

### DNP efficiencies on zeolites

**Fig. 1:**
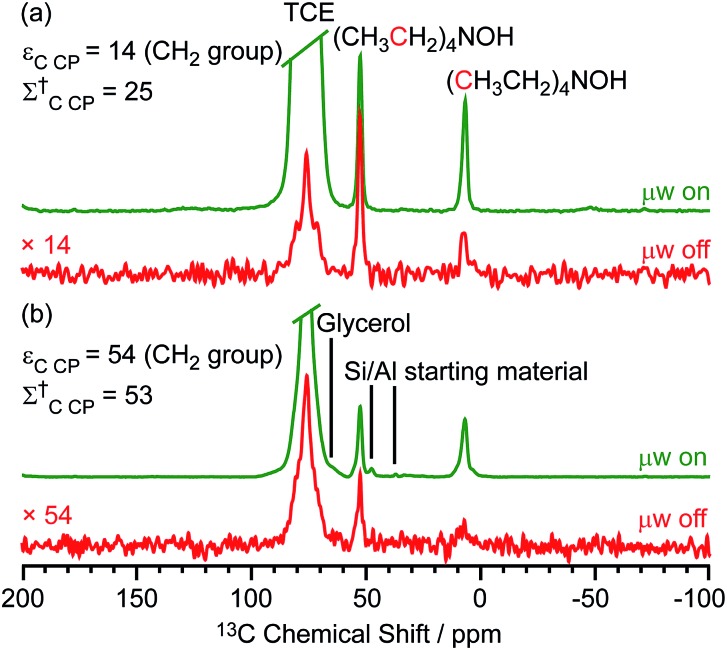
^13^C CP MAS DNP spectra of templated (a) M-β and (b) MMM-β with (μw on) (green) and without (μw off) microwave irradiation (red) at 9.4 T. TCE stands for 1,1,2,2-tetrachloroethane (solvent of impregnation).

Two different β-zeolites with different microstructures (microporous β-zeolite (M-β) and micro–meso–macroporous β-zeolite (MMM-β)^44^) were used in this study. The DNP efficiencies on these zeolites were initially explored on the pristine zeolites with organic templates. While moderate enhancements were only observed for M-β (*ε*_C CP_ = 14, *ε*_Si CP_ = 14), much larger enhancements of 54 on ^13^C (cross-polarization) CP and 72 on ^29^Si CP signals were obtained on MMM-β (Fig. 1, S2 and S3†), clearly showing the positive effects of introducing hierarchical porosities into zeolites. A more representative parameter to evaluate the DNP efficiency as compared with standard NMR at room temperature is the overall DNP gain *Σ*^†^ (see calculation details in the ESI†). This takes into account the increase of sensitivity from thermal Boltzmann distribution going from 298 K to 110 K (temperature at which the 9.4 T DNP with μw on data were recorded), signal attenuation due to the paramagnetic relaxation effects (bleaching) from the exogenous stable biradicals and cross-effect induced depolarization under magic angle spinning (MAS) conditions.^45^–^53^ *Σ*†C CP values of 25 and 53 for M-β and MMM-β and *Σ*†Si CP values of 34 and 97 for M-β and MMM-β, respectively, were obtained at 9.4 T (Tables S1 and S2 and Fig. 1, S2 and S3†). These large DNP gains *Σ*^†^ are clearly reflected in Fig. S2 and S3† by the much larger signal-to-noise ratios obtained from the μw on DNP ^29^Si spectra (recorded in seconds) *vs.* the room temperature ^29^Si MAS NMR spectra (acquired in tens of minutes).

The effects of paramagnetic centres on the NMR signals can be evaluated by the contribution factor *θ* and the increase of the NMR signal full width at half maximum (FWHM). The contribution factor *θ* corrects for the loss of signals by paramagnetic bleaching such that signals from nuclei close to the paramagnetic centres are removed beyond detection limits and by depolarization induced by the cross-effect under MAS conditions.^48^–^52^ A high contribution factor of 0.7 was obtained on the ^29^Si NMR signals for M-β (see Table S1†) while *θ* decreased to 0.5 for MMM-β (Table S2†), suggesting less overall signal loss in M-β than in MMM-β. The paramagnetic effect will also cause additional line-broadening of the NMR signals if the radicals are in proximity to the observed nuclei due to the faster nuclear transverse relaxations (
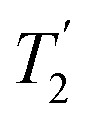
) induced by the radicals.^45^,^54^,^55^ Fig. S4 and S5† compare the ^13^C CP MAS NMR spectra of both M-β and MMM-β zeolites, respectively, in the presence and absence of radicals and no obvious change in FWHM is observed (as monitored by the CH_2_ resonance of the (CH_3_CH_2_)_4_NOH template). However, significant line broadening is observed when the zeolite is frozen in TCE at low temperature with the FWHM of the CH_2_ increasing from 100 Hz at room temperature to 210 Hz at 110 K (for M-β) and from 70 to 210 Hz (for MMM-β). This large line-broadening is attributed to the molecules being trapped in a variety of conformations by the low temperature and frozen solvent, leading to large inhomogeneous broadening (as generally observed in proteins).^47^,^54^,^56^ In this work, this broadening does not prevent the ^13^C resonances of the organic templates from being fully resolved.

The larger DNP enhancements in MMM-β *vs.* M-β can be attributed to the facilitated diffusion of both the large TEKPol radical (*d*_TEKPol_ ≈ 2 nm, with *d* referring to the length of molecule in a DFT optimized structure^34^) and 1,1,2,2-tetrachloroethane (TCE) solvent which is improved by the existence of uniform mesopores and macropores in MMM-β (mesopore and macropore sizes of 2.5–4.0 and 100–300 nm,^44^ respectively). Since the spin polarization transfer relies on the ^1^H solvent spin diffusion, more efficient polarization transfer in MMM-β is expected, which translates to larger enhancements. Additionally, a larger amount of radical solution is needed to wet the MMM-β zeolite which leads to higher electron spin concentration as confirmed by electron paramagnetic resonance (EPR) spin counting experiments (Table S3†).

### Fast detection of carbocations with DNP

**Fig. 2:**
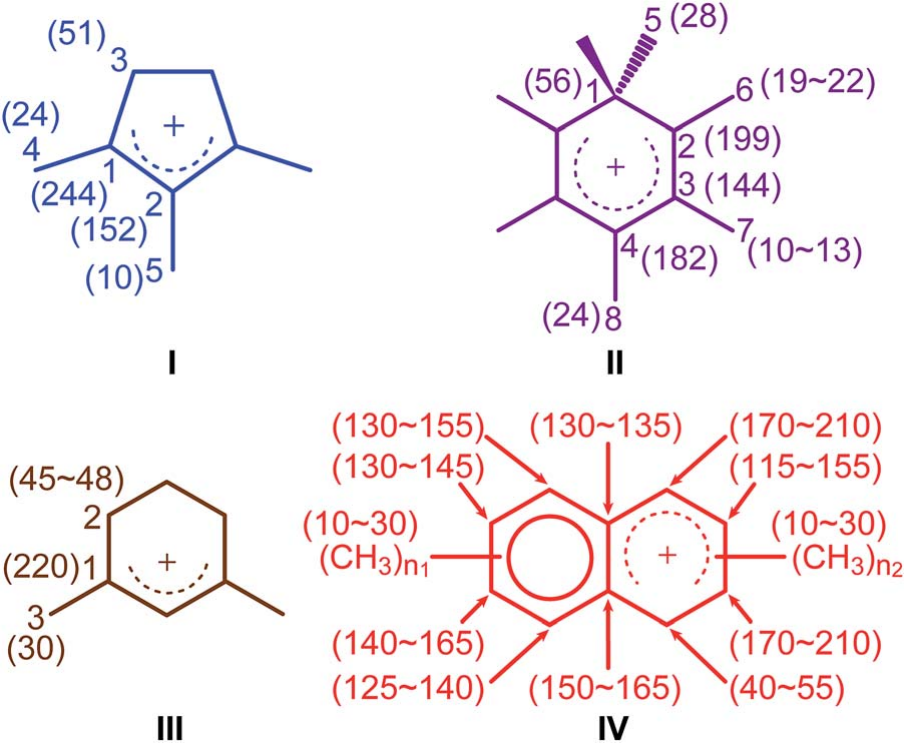
Carbocations identified in activated M-β and MMM-β coded with the same colors as their corresponding assignments and correlations in Fig. 3 and 4. Values in the parenthesis are ^13^C chemical shifts. *n*_1_ and *n*_2_ are the number of methyl groups with 3 ≤ (*n*_1_ + *n*_2_) ≤ 7.

The activated M-β was prepared by reacting M-β with ^13^CH_3_OH (see ESI† for experimental details). Both ^13^CH_3_OH and ^13^C_2_H_4_ were initially used to activate MMM-β; however the ^13^C_2_H_4_ activated MMM-β shows much stronger carbocation signals (Fig. S6†) and was therefore used in this work (unless otherwise specified). The chemical compatibility between the reactive cations, TCE and TEKPol solutions was investigated on these activated zeolites prior to DNP experiments. The M-β and MMM-β activated zeolites were impregnated with TCE and the ^13^C CP MAS spectra of both zeolites before and after impregnation with TCE are compared in Fig. S7.† The spectra show that, for both activated zeolites, the typical signals of carbocations (from 150 to 250 ppm), which structures identified in this work are shown in Fig. 2, and of the aromatics (120 to 150 ppm) remain unchanged after impregnation with TCE, indicating that the carbocations are well stabilised by confinement in pores. Note also that EPR spin counting experiments on the activated zeolites impregnated with the TCE/TEKPol biradical solution quantify the electron spin concentrations added (Table S3†) and illustrate the chemical compatibility of TCE/TEKPol with the carbocations and other carbon species formed in the activated zeolites. It is likely that the carbocations are mainly confined within the micropores of both β-zeolites (pore size < 1 nm), hence excluding the possibility of these cations reacting with bulky TEKPol (whose size is larger than the pore size of the micropores of the β-zeolite). It was demonstrated previously that immobilizing the reactive surface species inside a mesoporous support like MCM-41 (pore size of 2.5–3.0 nm) separates them from TEKPol and eliminates possible reactions between them while the polarization is still relayed by ^1^H spin diffusion of the solvent.^34^ This phenomenon is also responsible for the transfer of DNP polarization in the micropores of the Sn-β zeolite.^35^–^37^

**Fig. 3:**
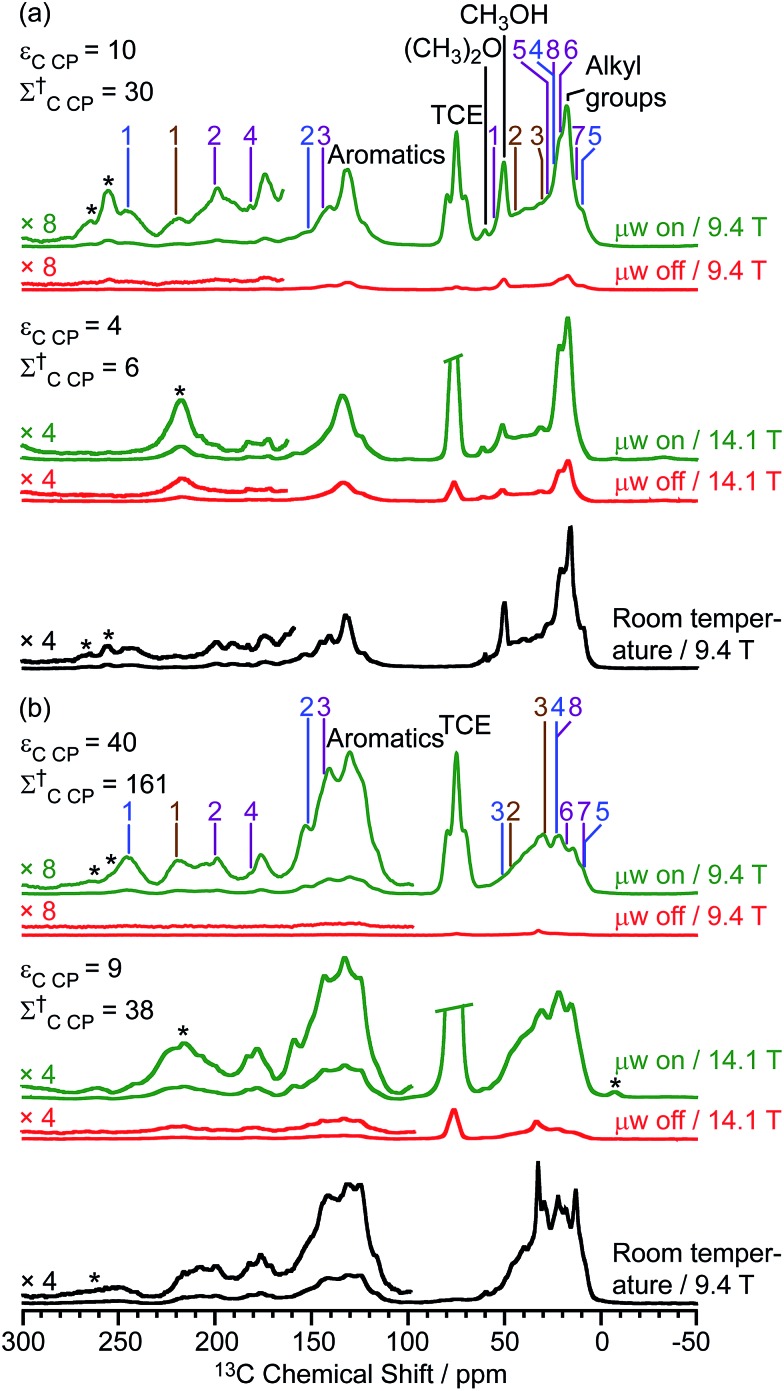
^13^C CP MAS DNP spectra at 9.4 T, 110 K and 14.1 T, 125 K and room temperature experiments at 9.4 T of (a) activated M-β and (b) activated MMM-β. The assignments of the different carbocations (except methylnaphthalenium ions **IV** for clarity) are given with the same colors as their structures in Fig. 2. The experimental times for the spectra of activated M-β are ≈7 minutes at 9.4 T DNP, ≈9 minutes at 14.1 T DNP and ≈52 minutes at room temperature at 9.4 T without DNP while for activated MMM-β they are ≈2 minutes at 9.4 T DNP, ≈6 minutes at 14.1 T DNP and ≈1036 minutes at room temperature at 9.4 T without DNP. *Σ*^†^ refers to the overall DNP gain and is calculated by comparing the DNP data to room temperature 9.4 T NMR spectra (see ESI†). All spectra were recorded at a MAS rate of 12.5 kHz with asterisks (*) denoting spinning sidebands.

The DNP-enhanced ^13^C CP MAS NMR spectra of the activated zeolites are shown in green in Fig. 3 and reveal ^13^C signal enhancements *ε*_C CP_ of 10 and 40 at 9.4 T for M-β and MMM-β, respectively. Under microwave irradiation at 9.4 T, the carbocation signals (150 to 250 ppm) can be clearly observed within minutes (experimental times of ≈7 minutes for M-β and ≈2 minutes for MMM-β) while, importantly, no carbocation signals emerge without microwave irradiation (red spectra). Other signals at around 0 to 50 ppm can be assigned to alkanes or alkyl groups from both aromatics and carbocations, while the two additional peaks at 50 and 60 ppm in activated M-β (Fig. 3a) correspond to methanol and dimethyl ether, respectively.^19^ The strong peak arising from the TCE solvent is located at about 75 ppm and does not interfere with the NMR signals of the adsorbed species (black spectra). The ^13^C signal enhancements above translate to overall DNP gains *Σ*†C CP of 30 and 161 for activated M-β (Table S4†) and MMM-β (Table S5†) zeolites, respectively. These gains correspond to a very significant reduction of experimental time compared to the standard experiments at room temperature, showing substantial DNP efficiency. Note the much better DNP efficiency on activated MMM-β than on activated M-β which validates the use of hierarchical pores. DNP experiments at 14.1 T were also recorded on both activated zeolites. While the ^13^C signal enhancements and DNP gains at 14.1 T are smaller than those at 9.4 T (Fig. 3), as expected from the inverse field-dependence of the DNP enhancement with cross-effect DNP,^29^,^30^ the carbocations can still be observed but no obvious increase in resolution is observed presumably due to strong inhomogeneous broadening and increase in chemical shift dispersion.

Large contribution factors (*θ* values ranging between 0.7 and 0.8) were obtained for the ^13^C signals of activated M-β and MMM-β (Tables S4 and S5†). These results indicate less signal loss by paramagnetic bleaching and depolarization in the two activated zeolites. The FWHM of the CH_3_OH signal in activated M-β was measured and used to monitor the changes of the ^13^C lineshapes in the presence of TEKPol and TCE and at low temperatures. Fig. S8† shows that adding TEKPol only increases the linewidth slightly by about 8% (from 455 to 490 Hz). However, a significantly larger line broadening is observed when activated M-β is frozen at low temperatures with the FWHM of CH_3_OH increasing by 25% (from 365 Hz at room temperature to 455 Hz at 110 K), resulting in a slight loss of resolution (*e.g.* the double peaks at 145 ppm and at 21 ppm are not resolved anymore, Fig. S8†). A similar observation is made in activated MMM-β (see Fig. S9†) with a clear loss of resolution of the signals arising from the alkyl groups. The results indicate inhomogeneous broadening due to molecules being trapped in a variety of conformations as the main contribution to the line-broadening, as on the zeolites with templates.^47^,^54^,^56^

### Structure identification of the carbocations

**Fig. 4:**
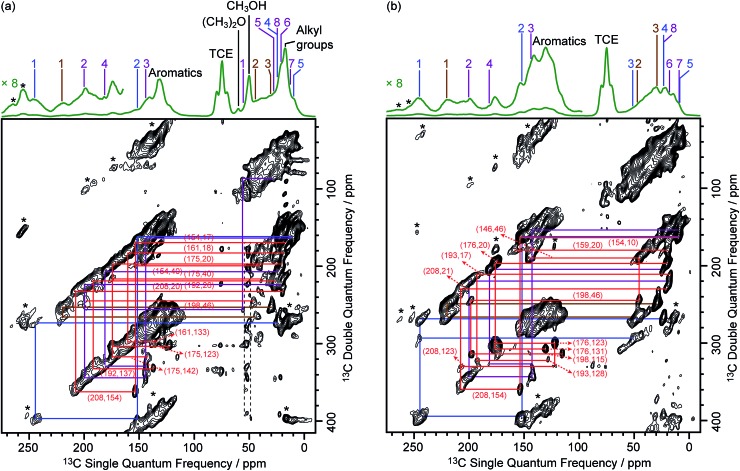
DNP enhanced 2D ^13^C–^13^C refocused INADEQUATE spectra of (a) activated M-β and (b) activated MMM-β. Data were recorded at *B*_0_ = 9.4 T and a MAS frequency of *v*_r_ = 12.5 kHz. Experimental times for (a) and (b) are ≈20 and 14 hours, respectively. The correlations and spectral assignments are coded with the same colors as their corresponding carbocations in Fig. 2. Signals in the black dashed box in (a) correspond to *t*_1_ noise. Correlations corresponding to naphthalenium ions (**IV**) are shown with the ^13^C chemical shifts in the single quantum dimension of the two correlated carbon atoms given in parenthesis. Enlarged figures are shown in Fig. S10 and S11.† Asterisks (*) denote spinning sidebands.

To identify the molecular structures of the confined carbon species, a 2D ^13^C–^13^C refocused INADEQUATE (Incredible Natural Abundance DoublE QUAntum Transfer Experiment),^41^ based on scalar *J* couplings and providing ^13^C–^13^C through-bond correlations, was performed. Spectra are shown in Fig. 4: three single ring carbocations can be identified which are trimethylcyclopentenyl cation **I**, heptamethylbenzenium cation **II** and dimethylcyclohexenyl cation **III**, confirming the previously postulated structures.^16^,^19^,^57^,^58^ More explicitly, the structure of **I** is obtained through the following correlations: C1(**I**) (244 ppm)–C2(**I**) (152 ppm), C1(**I**) (244 ppm)–C3(**I**) (51 ppm), C1(**I**) (244 ppm)–C4(**I**) (24 ppm) and C2(**I**) (152 ppm)–C5(**I**) (10 ppm) (see Fig. S10 and S11† for the derivation of **II** and **III** from these data). Note that cation **I** was previously identified as an intermediate over ZSM-5 (ref. 16 and 23) and SAPO-34 (ref. 58), but has only been postulated over β zeolites,^19^ with the data presented here therefore confirming its presence. These five- and six-membered ring cations suggest that both the paring and side-chain catalytic cycles may exist in the β zeolite for the conversion of methanol to hydrocarbons, and the experimentally structural identification of these cations here provides more directly spectroscopic support for the previously proposed mechanisms.^15^,^16^,^19^,^59^

Neutral methylnaphthalenes have been identified by GC-MS as part of the hydrocarbon pool species in the β zeolite;^19^,^59^,^60^ however observation of their active cationic counterparts (methylnaphthalenium ions) and determination of those cations' structures have only been elusive so far even when ^13^C solid-state NMR was previously deployed.^15^,^16^,^19^ The extra sensitivity obtained with DNP permits the detection of additional ^13^C correlations (shown in red) involving lower field signals at 208 and 176 ppm to be also resolved (Fig. 4, S10 and S11†). These characteristic peaks have been observed in previous liquid-state ^13^C NMR studies of methylnaphthalenium ions bearing up to four methyl substituents and are obtained by protonation of methylnaphthalenes by magic acids.^61^,^62^

The DNP enhanced 2D ^13^C–^13^C NMR correlation experiments provide direct support for identifying the structure of these methylnaphthalenium ions. Characteristic correlations in the single quantum (horizontal) dimension at, for example, 208–154 ppm and 176–131 ppm are assigned to ring carbons while those at 208–21 and 176–20 ppm correspond to bonds between ring carbons and methyl substituents, and enable methylnaphthalenium ions with structure **IV** to be proposed (Fig. 2). The range of connectivities shown in Fig. 4 demonstrates that more than one methylnaphthalenium ion is present and highlights the actual complexity of these species in activated zeolites (Fig. 4).

These results confirmed previous computational^63^ and UV-vis spectroscopy^64^ studies that postulated the presence of methylnaphthalenium ions and methylnaphthalenes. This is also in agreement with previous GC-MS data which suggest that methylnaphthalenes with 3 to 7 methyl groups exist in the β zeolite.^59^,^60^ These species can act as both active hydrocarbon pool species to convert methanol into targeted hydrocarbons,^60^,^63^–^65^ and as coke precursors leading to zeolite deactivation.^63^,^64^

Correlations arising from neutral carbon species such as aromatics and alkanes^19^ are also shown in Fig. S12 and S13† from which structures such as methylnaphthalenes,^60^,^66^ hexamethylmethylenecyclohexadiene,^59^,^67^ hexamethylbenzene,^66^,^68^ *etc.* could be possibly derived.

It is noteworthy that fairly similar cyclic hydrocarbon pool species were identified in both ^13^CH_3_OH activated M-β and ^13^C_2_H_4_ activated MMM-β zeolites (Fig. 4). A reasonable route for the formation of the cyclic hydrocarbon species can be proposed here and starts with the initial C–C bond formation from C1 reactants such as CH_3_OH and its derivatives to produce ethylene first^69^ which can then produce cyclic hydrocarbon species *via* polymerization and cyclization (Fig. S14†).

In addition to the scalar coupling based INADEQUATE discussed above, a 2D ^13^C–^13^C Proton Driven Spin Diffusion (PDSD) Dipolar Assisted Rotational Resonance (DARR) MAS correlation experiment on activated MMM-β (Fig. S15†) was also obtained. In this experiment, cross peaks arise from spatial proximities of the species or chemical exchange.^70^–^72^ Using a short mixing time of 30 ms, intramolecular correlations are observed,^47^ and indeed, correlations from the directly bonded ^13^C nuclei from the same carbocations are identified in Fig. S15,† which is consistent with the INADEQUATE results. A very small number of correlations from non-bonded ^13^C nuclei can also be observed in the PDSD DARR spectrum (for example, C1(**I**) (244 ppm)–C5(**I**) (10 ppm)), reinforcing the INADEQUATE spectral interpretation and the identified carbocations (Fig. 2).

### Investigation of the reaction process

**Fig. 5:**
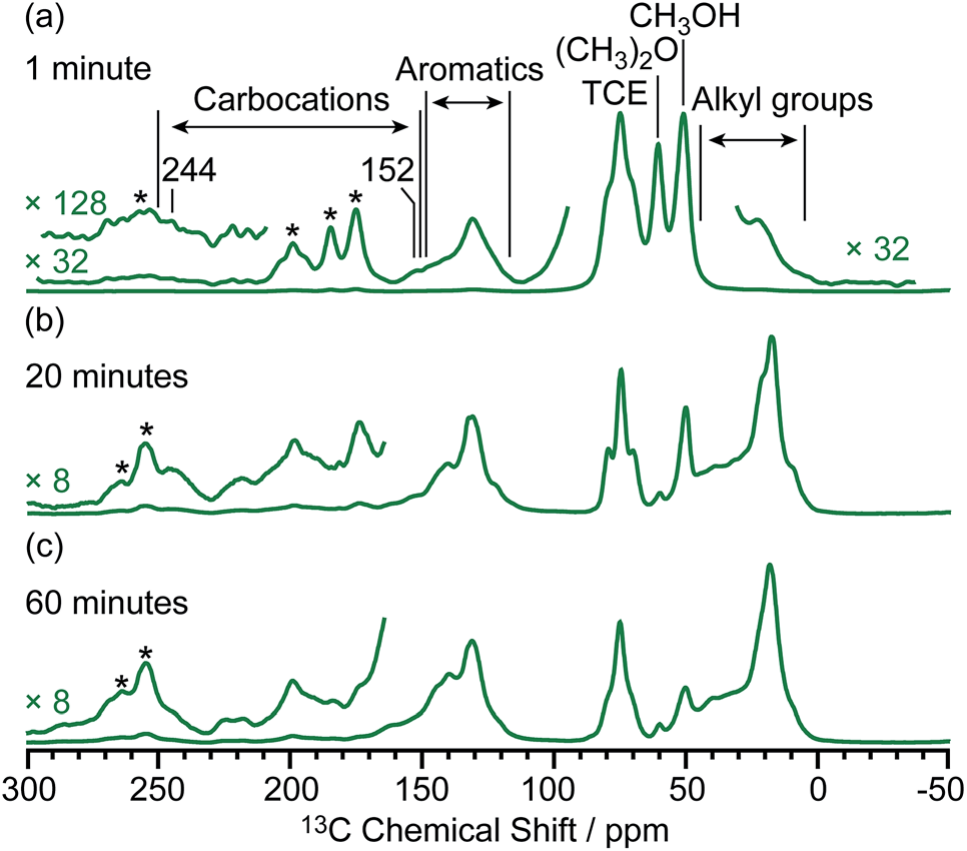
μw on ^13^C CP MAS DNP spectra of M-β activated for (a) one minute, (b) 20 minutes and (c) 60 minutes. Spectra were recorded at 9.4 T and at a MAS rate of 12.5 kHz. Asterisks (*) denote spinning sidebands. Additional spectra at different MAS rates of M-β activated for 20 minutes are shown in Fig. S16.†

Based on the structural determination of the carbon species and the largely increased sensitivity provided by DNP, we further investigated the reaction process by monitoring *ex situ* the evolution of the DNP enhanced ^13^C NMR spectra of activated M-β zeolites with variable ^13^CH_3_OH activation times (Fig. 5). The spectra show that even after a very short activation of only one minute, signals from aromatics and carbocations (*e.g.* resonances at 244 and 152 for **I**) are detected. The results demonstrate that the hydrocarbon pool species can be formed at a very early stage of the induction period of the MTH reaction. As activation times increased, the signals of aromatics and carbocations increase in intensity pointing out to the accumulation of these species. The spectrum of M-β activated for 60 minutes is fairly similar to the one after 20 minutes, suggesting that the components of the hydrocarbon pool species are steady over these reaction times.

### Investigation of host–guest interaction using ^29^Si{^13^C} REDOR (Rotational Echo DOuble Resonance)^42^

**Fig. 6:**
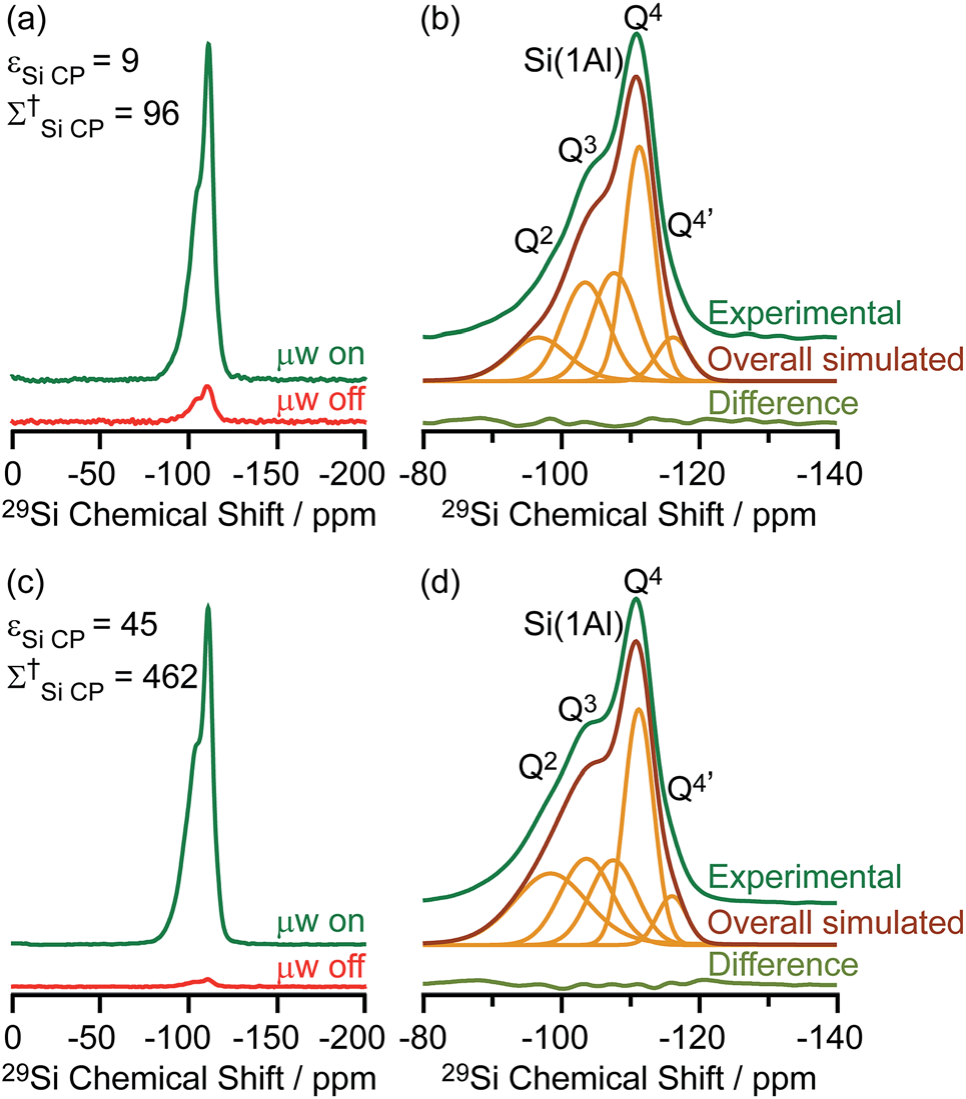
(a and c) ^29^Si CP MAS DNP spectra and (b and d) μw on experimental ^29^Si CP MAS DNP spectra with spectral deconvolution, overall simulated lineshape and difference between experimental and simulated spectra of activated M-β (a and b) and activated MMM-β (c and d). All spectra were recorded at 9.4 T and at a MAS rate of 8 kHz. *Σ*^†^ refers to the overall DNP gain and is calculated by comparing the DNP data to room temperature 9.4 T NMR spectra (see ESI†).

The DNP enhanced ^29^Si CP MAS spectra of activated M-β and activated MMM-β are shown in Fig. 6 and both show multiple resonances at –96, –103, –107, –111 and –116 ppm which are characteristic of the (SiO)_2_Si(OH)_2_ (Q^2^), (SiO)_3_SiOH (Q^3^), Si(OSi)_3_(OAl) (Si(1Al)), Si(OSi)_4_ (Q^4^) and the crystallographically inequivalent Si(OSi)_4_ (Q^4^′) sites, respectively, of which the Si(1Al) sites contribute to the Brønsted acid sites.^73^,^74^ *ε*_Si CP_ DNP enhancement of 9 and overall DNP gain *Σ*†Si CP of 96 for M-β (Fig. 6a) and *ε*_Si CP_ of 45 and *Σ*†Si CP of 462 for MMM-β (Fig. 6c) were obtained.

**Fig. 7:**
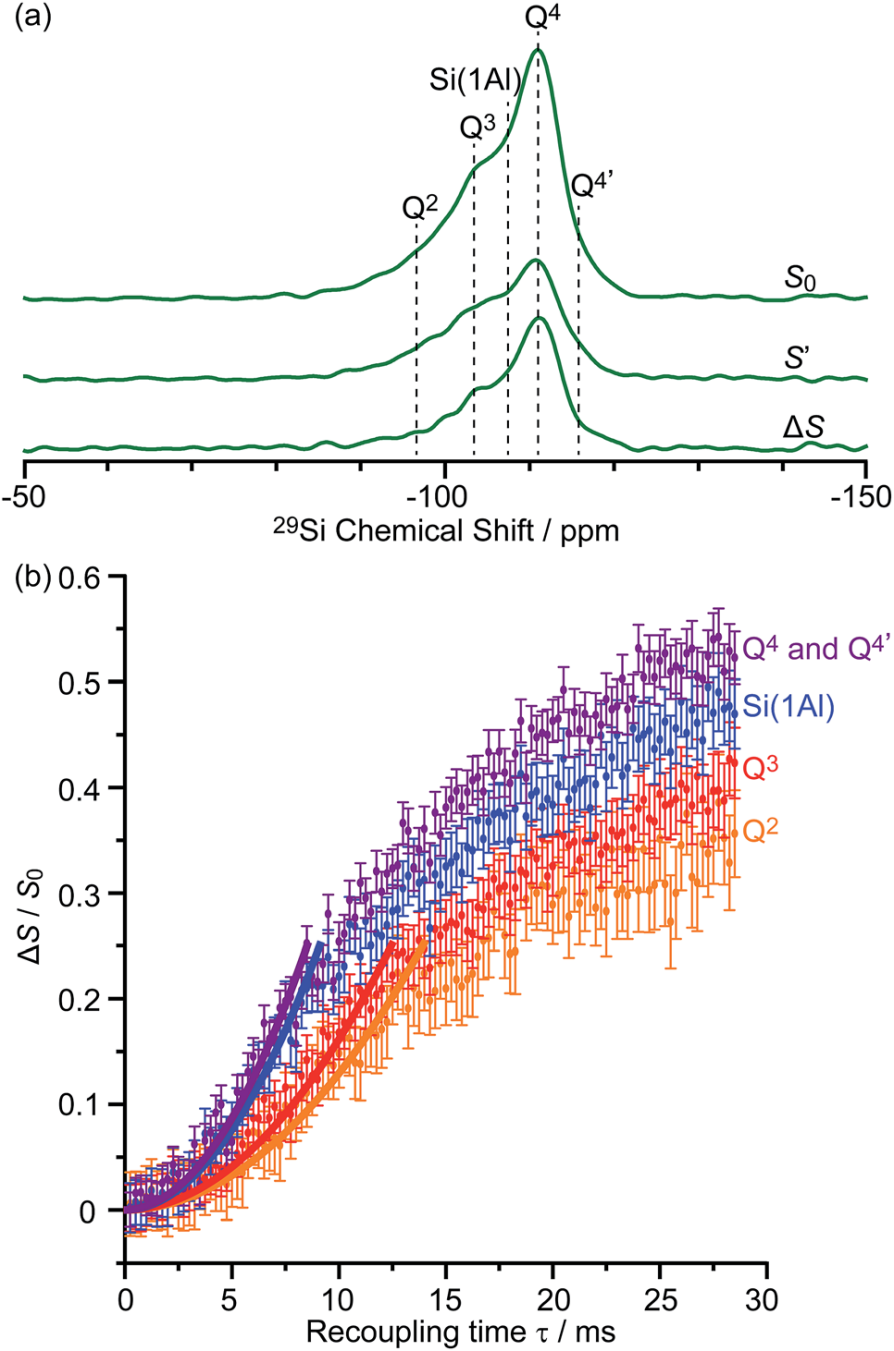
(a) DNP enhanced ^29^Si CP spin echo spectrum (*S*_0_) and ^29^Si{^13^C} REDOR spectrum (*S*′) with the reintroduction of dipolar couplings at a recoupling time of 28.5 ms. Δ*S* is the difference spectrum *S*_0_ – *S*′. Spectra were recorded at 9.4 T on activated M-β. (b) ^29^Si{^13^C} REDOR fraction Δ*S*/*S*_0_ as a function of the recoupling time up to 28.5 ms. The experimental time is ≈15 hours. The solid lines are the best-fit of the REDOR curves up to a Δ*S*/*S*_0_ of 0.25 using a first-order approximation and eqn (S4) in the ESI.† 75 The ^29^Si–^13^C dipolar coupling values are given in Table 1. The vertical error bars correspond to the error analysis as given in Section 3 in the ESI.† The REDOR curves for each ^29^Si site are also shown separately in Fig. S18.†

These sensitivities permit the fast collection of ^29^Si detected ^29^Si{^13^C} REDOR experiments with high signal-to-noise (S/N) ratios which would otherwise be extremely time consuming. These experiments reintroduce the ^29^Si–^13^C dipolar couplings under MAS^42^ allowing the spatial proximities between the confined carbon species and surface sites of the zeolite to be quantitatively probed. At a recoupling time of 28.5 ms for M-β (Fig. 7a) and 30 ms for MMM-β (Fig. S17†), the ^13^C dephased ^29^Si detected signals *S*′ show significant reduction in intensities compared with the spin echo signal *S*_0_, demonstrating spatial proximities between ^29^Si and ^13^C nuclei. The high S/N ratios permit small differences in the evolutionary pattern of the REDOR fraction Δ*S*/*S*_0_ as a function of the recoupling times for individual Si sites to be distinguished. The REDOR curves for different Si sites are overlaid in Fig. 7b (for M-β) and S17† (for MMM-β) and demonstrate clear differences between these Si sites. Separate figures for each Si site can also be found in Fig. S18 and S19.†

Considering the number of retained carbon species and their unknown geometries in the zeolites, a geometrically independent REDOR curve model which only requires data at short dipolar evolution times (Δ*S*/*S*_0_ up to 0.25)^75^ was used to fit the REDOR data (see ESI† for further details) and the results are summarized in Table 1. By further assuming a ^29^Si–^13^C single spin pair model, an estimation of the ^29^Si–^13^C dipolar coupling strengths and distances is also given.

**Table 1:** ^29^Si–^13^C dipolar coupling strengths *D* and distances *r* in activated M-β and MMM-β obtained from the ^29^Si{^13^C} REDOR experiments (see ESI for the fitting procedure and description of the fitting model)

Zeolite	^29^Si sites	∑*D*_i_^2^/Hz^2^	*D* ^*a*^/Hz	*r* ^*a*^/Å
Activated M-β	Q^2^	1200 ± 400	35 ± 7	5.6 ± 0.4
	Q^3^	1500 ± 500	39 ± 7	5.4 ± 0.3
	Si(1Al)	2800 ± 800	53 ± 8	4.8 ± 0.3
	Q^4^ + Q^4^′	3200 ± 800	57 ± 8	4.7 ± 0.3
Activated MMM-β	Q^2^	65 ± 25	8 ± 2	9.1 ± 0.7
	Q^3^	105 ± 40	10 ± 2	8.4 ± 0.7
	Si(1Al)	155 ± 40	12 ± 2	7.8 ± 0.4
	Q^4^ + Q^4^′	235 ± 45	15 ± 2	7.3 ± 0.3

^*a*^Assuming a simplified ^29^Si–^13^C single spin pair model.

The ^29^Si{^13^C} REDOR data (Table 1) show that both Q^4^ and Si(1Al) sites have the strongest interaction with the hydrocarbon pool species in activated M-β with ∑*D*_i_^2^ being 3200 ± 800 Hz^2^ and 2800 ± 800 Hz^2^, respectively. This major contribution to the ^29^Si{^13^C} REDOR curves is expected as neutral aromatic species are the main hydrocarbon pool species, as shown in the ^13^C CP spectra, and interact more strongly with the zeolite frameworks. The similar interaction between the hydrocarbons and both Q^4^ and Si(1Al) sites also suggests that the van der Waals interaction with the zeolite framework dominates the adsorption of these hydrocarbon pool species within the micropores (known as the confinement effects)^76^ and that, surprisingly, there is no evidence for preferential interaction with the Brønsted acid sites of the zeolites. The zeolite deactivation during the MTH process is therefore likely due to the accumulation of aromatics in the channels of the zeolite and their further growth to form polycyclic cokes blocking the reactants' accesses to the catalytic acid sites supporting previous computational studies.^76^,^77^ The quantitative information provided here also promotes understanding of the nature of the previously proposed supramolecular reaction centers^78^ and yields structural constraints (distance of around 4.8 Å) between the hydrocarbon pool species and zeolite frameworks. Table 1 also shows that the Q^2^ and Q^3^ sites have much weaker interaction with the hydrocarbon pool species suggesting that these silanol defects are mainly located at the external surface.

Comparison of the ^29^Si{^13^C} REDOR data for both activated zeolites (Table 1) revealed that the Si sites show much weaker interaction with the hydrocarbon pool species in MMM-β than in M-β. We ascribe this phenomenon to the presence of the mesopores in MMM-β which weaken the confinement effects and suggest that some carbon species should predominantly locate in the mesopores.

Note that the ^29^Si{^13^C} REDOR results do not exclude the possibility that carbocations have strong interaction with the Brønsted acid sites, likely *via* the formation of ion-pair complexes,^79^ since the ^29^Si{^13^C} REDOR experiments measure the overall dipolar coupling to ^29^Si from ^13^C spins of different molecules while not distinguishing the source of the contribution.

## Conclusions

In summary, we demonstrate that a small concentration of carbocations confined in zeolites can be detected within minutes by DNP enhanced multinuclear NMR spectroscopy. The large DNP signal enhancements enable acquisition of two-dimensional ^13^C–^13^C NMR correlation experiments in hours which would otherwise take days or even weeks without DNP. These correlations permit the identification of a series of five- and six-membered ring carbocations serving as intermediates in the MTH reaction. In particular, methylnaphthalenium ions are identified and reinforce their importance as hydrocarbon pool species for the formation of targeted hydrocarbon products and coke precursors leading to zeolite deactivation. Additionally, the host–guest interaction between various silicon sites of zeolites and hydrocarbon pool species is quantitatively determined *via* DNP enhanced ^29^Si{^13^C} REDOR experiments, which indicate that van der Waals interaction with the zeolite frameworks dominates the adsorption of the majority hydrocarbon pool species, suggesting accumulation of these species in the channels and leading to zeolite deactivation. Finally, we show that introducing hierarchical pores into zeolites is a promising way to improve DNP efficiency on this type of materials. The implications of this strategy to tackle the understanding of hierarchically structured porous materials of considerable interest in catalysis, gas adsorption, sensing, *etc.*^43^,^80^ are potentially very large.

## Author contributions

D. X. and F. B. designed the project, conducted the DNP experiments, analysed the data and wrote the manuscript with inputs from all co-authors. S. X. prepared the activated zeolites, contributed to the DNP experiments and data analysis. N. B., S. P. and F. A. aided in carrying out the DNP experiments. F. A. performed the EPR spin counting experiments. L. C. and B. S. prepared the MMM-β zeolite. All authors discussed the results. D. X. and S. X. contributed equally.

## Conflicts of interest

There are no conflicts to declare.

## Supplementary Material

Supplementary information
