# Supplementary material for: Fast detection and structural identification of carbocations on zeolites by dynamic nuclear polarization enhanced solid-state NMR
Source: Chem Sci. 2018 Oct 2;9(43):8184–93. doi: 10.1039/c8sc03848a (PMC6254210; doi:10.1039/c8sc03848a)
Supplement: Supplementary file 1 [file SC-009-C8SC03848A-s001.pdf]

Electronic Supplementary Material (ESI) for Chemical Science.
This journal is © The Royal Society of Chemistry 2018

Supplementary Information for:

Fast Detection and Structural Identification of Carbocations on Zeolites by Dynamic Nuclear Polarization Enhanced Solid-State NMR

Dong Xiao+,[abc] Shutao Xu+,[d] Nick J. Brownbill,[c] Subhradip Paul,[e] Li-Hua Chen,[f] Shane Pawsey,[g] Fabien Aussenac,[h] Bao-Lian Su,[fi] Xiuwen Han,[a] Xinhe Bao,[a] Zhongmin Liu,[ad] and Frédéric Blanc*[cj]

a State Key Laboratory of Catalysis, Dalian Institute of Chemical Physics, Chinese Academy of Sciences, 457 Zhongshan Road, Dalian 116023, China

b University of Chinese Academy of Sciences, Beijing 100049, China

c Department of Chemistry, University of Liverpool, Crown Street, Liverpool, L69 7ZD, United Kingdom

d National Engineering Laboratory for Methanol to Olefins, Dalian National Laboratory for Clean Energy, Dalian Institute of Chemical Physics Chinese Academy of Sciences, Dalian 116023, China

e DNP MAS NMR Facility, Sir Peter Mansfield Imaging Centre, University of Nottingham, Nottingham NG7 2RD, United Kingdom

f State Key Laboratory of Advanced Technology for Materials Synthesis and Processing, Wuhan University of Technology, 122 Luoshi Road, 430070, Wuhan, China

g Bruker BioSpin Corporation, 15 Fortune Drive, Billerica, Massachusetts 01821, United States

h Bruker BioSpin, 34 rue de l'Industrie BP 10002, 67166 Wissembourg Cedex, France

i CMI (Laboratory of Inorganic Materials Chemistry), University of Namur, 61 rue de Bruxelles, B-5000 Namur, Belgium

j Stephenson Institute for Renewable Energy, University of Liverpool, Crown Street, Liverpool L69 7ZD, United Kingdom

+ These authors contributed equally to this work.

* To whom correspondence should be addressed (F.B.).

E-mail: frederic.blanc@liverpool.ac.uk.

Table of Contents

Sections	Contents	Page No
S1	Experimental methods	3
S2	Measurement of overall DNP sensitivity enhancement factor $\Sigma^{\dagger}$	6
S3	Process for extraction the ^{29}Si-^{13}C dipolar couplings from the ^{29}Si{^{13}C} REDOR data	7
S4	Additional tables	8
S5	Additional figures	14
S6	References	33

S1. Experimental methods

Preparation of activated zeolites. Two different β-zeolites with organic templates and different microstructures (microporous β-zeolite (M-β) and micro-meso-macroporous β-zeolite (MMM-β)[1]) were used. The β-zeolite has 12-ring window of 7.7 × 6.7 Å and 5.6 × 5.6 Å viewed along [100] and [001], respectively. The M-β zeolite templated with tetraethylammonium hydroxide was obtained from China University of Petroleum and has a Si/Al ratio of 13 (framework Si/Al ratio ≈ 24),[2] while MMM-β (framework Si/Al ratio ≈ 34, see Table S6, Figure S20) with tetraethylammonium hydroxide and glycerol templates was synthetized following a literature procedure.[1] Both zeolites were then calcined in air at 550 °C for 6 h to remove the templates, ion-exchanged in 1.0 M NH_4NO_3 aqueous solution at 80 °C three times and then subsequently recalcined at 550 °C for 4 h to yield the H-type zeolites. The zeolites were dehydrated at 500 °C under a continuous flow of He flow at 25 $mL\cdot min^{-1}$ for 2 h in a fixed-bed quartz tubular reactor followed by a decrease in temperature to the reaction temperature as follow. M-β was then reacted for 20 minutes (unless otherwise specified) with $^{13}CH_3OH$ (99 atom % ^{13}C, Sigma-Aldrich) with a weight hourly space velocity (WHSV) of 2 h^{-1} at 275 °C while MMM-β was reacted for 20 minutes with either $^{13}CH_3OH$ at 300 °C or $^{13}C_2H_4$ (99 atom % ^{13}C, Sigma-Aldrich) at 275 °C with a WHSV of 2 h^{-1}. The reaction was then quenched by immersing the reactor into liquid N_2 and transferred to the N_2 glove box at room temperature where the samples were stored.

The carbocation concentrations of activated M-β and activated MMM-β are in the order of 0.002 - 0.01 mmol/g based on the ^{13}C NMR spectra and previous work,[2] while the one of activated MMM-β is much lower.

Chemical compatibility between carbocations and 1,1,2,2-tetrachloroethane (TCE) solvent. The activated zeolites (30 to 50 mg) were mixed with the halogenated solvent TCE (about 50 μL) by incipient wetness impregnation. Room temperature ^{13}C cross-polarization (CP) magic angle spinning (MAS) spectra were recorded at 14.1 T before and after mixing with TCE.

Solid-State NMR. NMR spectra were recorded on Bruker Avance III spectrometers (9.4 T and 14.1 T) using 4 mm HXY probes in double resonance mode.

^{13}C CP MAS experiments at 9.4 T were performed at a MAS rate of 12.5 kHz, while ^{29}Si CP MAS experiments were performed at a MAS rate of either 8 kHz (for M-β with template, MMM-β with template and activated M-β) or 10 kHz (for activated MMM-β). All ^{1}H pulses and SPINAL-64 heteronuclear decoupling[3] were performed at radio-frequency (rf) field amplitude of 83 kHz. ^{13}C CP MAS and ^{29}Si CP MAS experiments were obtained with a ^{13}C rf field of 51 kHz and a

^{29}Si rf field of 53 and 45 kHz at MAS rates of 8 and 10 kHz, respectively, while the ^{1}H rf field was ramped to obtain maximum signals at approximately 60 kHz.[4]

^{13}C CP MAS experiments at 14.1 T were performed at a MAS rate of 12 kHz. All ^{1}H pulses and SPINAL-64 heteronuclear decoupling were performed at a rf field of 54 kHz. ^{13}C CP MAS experiments were obtained with ^{13}C rf field of 79 kHz while the ^{1}H rf field was ramped at approximately 54 kHz.

The directly excited ^{29}Si MAS NMR spectrum of the H-type MMM-β zeolite was acquired at 9.4 T at a MAS rate of 10 kHz and with ^{1}H SPINAL-64 high-power decoupling[3] at a rf field of 83 kHz. ^{29}Si pulses were obtained with rf field of 70 kHz. A recycle delay of 5 × T_1 = 65 s was used where T_1 is the ^{29}Si longitudinal relaxation time constant.

DNP MAS NMR. Samples for DNP experiments were prepared by incipient wetness impregnation of the solids (typically 30 mg) with 40 μL of 20 mM of nitroxide biradicals TEKPol[5] in TCE[5,6] which is currently one of the most efficient polarizing matrix for cross-effect DNP at 9.4-14.1 T.[35,36] The freshly prepared mixtures were then packed into sapphire rotor which was then inserted into the pre-cooled DNP probe.

All DNP experiments were performed on a commercial 9.4 T Avance III DNP solid-state NMR spectrometer equipped with a 263 GHz gyrotron and on a 14.1 T Avance III DNP NMR spectrometer equipped with a 395 GHz gyrotron, using a low temperature 3.2 mm HXY probe in double or triple resonance mode. All the ^{13}C and ^{29}Si CP MAS experiments were performed with ^{1}H pulses and SPINAL-64 decoupling[3] at a rf field of 100 kHz and a recycle delay of 1.3 × $\tau_{DNP}(^{1}H)$ where $\tau_{DNP}(^{1}H)$ is the DNP build-up time. A ^{1}H pre-saturation block of 100 pulses separated by 1 ms was applied.

^{13}C and ^{29}Si CP MAS experiments at 9.4 T were performed at MAS rates of 12.5 and 8 kHz, respectively, and at T ≈ 110 K (calculated through T_1 of ^{79}Br in KBr[7] for which a very small amount was added to the rotor). The ^{13}C CP experiments were obtained with ^{13}C rf field at 62.5 kHz matched to a ramped ^{1}H rf field at approximately 100 kHz while ^{29}Si CP experiments were obtained with ^{29}Si rf field at 62.5 kHz matched to a ramped ^{1}H rf field at approximately 102 kHz. The rf field of the ^{13}C and ^{29}Si pulses was set to 62.5 kHz in the ^{13}C-^{13}C z-filtered refocused INADEQUATE (Incredible Natural Abundance DoublE QUAntum Transfer Experiment)[8], ^{13}C-^{13}C PDSD (Proton Driven Spin Diffusion)[9–11], and ^{29}Si{^{13}C} REDOR (Rotational Echo DOuble Resonance)[12] experiments (pulse programs are shown in Figure S1). The rotor synchronized τ delays in the INADEQUATE experiments were optimized to about 1.6 ms. The z-filter duration for activated M-β and MMM-β was 0.8 ms and 0.4 ms, respectively, during which ^{1}H continuous

wave decoupling at ^{1}H rf field of 12.5 kHz was used.[13] In the ^{13}C-^{13}C PDSD experiments, a 30 ms mixing time was used. DARR (Dipolar Assisted Rotational Resonance) sequence was applied during the mixing period with ^{1}H rf field equal to MAS rate at 12.5 kHz. In the ^{29}Si{^{13}C} REDOR experiments, recoupling times up to 28.5 ms (for activated M-β) and 30 ms (for activated MMM-β were used. The experimental spectral lineshapes and dephasing curves from the REDOR experiments were simulated with the DMFIT[14] fitting software and using MatLab R2016a, respectively.

^{13}C CP MAS experiments at 14.1 T were performed at a MAS rate of 12.5 kHz and at T ≈ 125 K. The spectra were obtained with a ^{13}C rf field at 62 kHz matched to a ramped ^{1}H rf field at approximate 90 kHz.

EPR spin counting. Electron paramagnetic resonance (EPR) experiments were recorded at room temperature on the DNP samples packed into 3.2 mm sapphire NMR rotors using an X-band Bruker Biospin EMX Nano spectrometer operating at X-band (9.6 GHz). The error on the EPR spin counting experiments was estimated to be about ±30%.[15,16] The concentration of electron spins in the DNP samples was also calculated based on the amount of zeolite and radical solution added. The error was estimated to be about ±75%, considering the evaporation of TCE solvent, error in radical concentration in solution, error in sample mass, etc.[17]

S2. Measurement of overall dynamic nuclear polarization (DNP) sensitivity enhancement factor $\Sigma^\dagger$[18–26]

The overall DNP sensitivity enhancement factor $\Sigma^\dagger$ takes into account the sensitivity improvement from low temperature thermal Boltzmann enhancement (DNP with μw (microwave) on), signal attenuation due to the paramagnetic relaxation effects (bleaching) from the exogenous stable biradicals and cross-effect induced depolarization at MAS condition,[21–25] and time differences between the DNP enhanced nuclear magnetic resonance (NMR) experiments and standard NMR experiments at room temperature. The following experiments are used to determine the factor $\Sigma^\dagger$:

(a) μw on DNP enhanced NMR at low temperature (approx. 110 K) of the zeolites impregnated by 20 mM of TEKPol[5] in TCE[5,6],

(b) μw off DNP NMR at low temperature (approx. 110 K) of the zeolites impregnated by 20 mM of TEKPol in TCE,

(c) Room temperature NMR of the neat zeolites,

(d) μw off DNP NMR at low temperature (approx. 110 K) of the zeolites impregnated with TCE and at the same field as in (a).

The equation to calculate $\Sigma^\dagger$ is as follows:

$$\Sigma^\dagger = \left(\frac{\Lambda_{RT}}{\Lambda_{DNP}}\right)\left(\frac{T_{RT}}{T_{DNP}}\right)\left(\frac{(S/N)_{DNP}\, m_{RT}}{(S/N)_{RT}\, m_{DNP}}\right)\sqrt{\frac{NS_{RT}\, T_{1,\,RT}}{NS_{DNP}\, \tau_{DNP}}}\,\theta \qquad \text{Equation S1}$$

where Λ, T, S/N, m, NS, $T_{1,\,RT}$, τ_{DNP} and θ are the full width at half maximum (FWHM), sample temperature, signal-to-noise ratio, mass of sample used, number of scans accumulated, ^{1}H longitudinal relaxation time constant at room temperature, DNP polarization build-up time and contribution factor,[23,25] respectively. The subscripts DNP and RT represent parameters taken from experiments (a) and (c), respectively. The contribution factor is calculated as:

$$\theta = \frac{1}{\varepsilon}\left(\frac{(S/N)_{DNP}\, m_{LT}}{(S/N)_{LT}\, m_{DNP}}\right)\sqrt{\frac{NS_{LT}}{NS_{DNP}}} \qquad \text{Equation S2}$$

where ε is the DNP signal enhancement between (a) and (b), $(S/N)_{LT}$, m_{LT}, and NS_{LT} are signal-to-noise ratio, mass of sample and number of scans accumulated for (d), respectively.

In this paper, $\Sigma^\dagger$ values were calculated for M-β and MMM-β with templates and activated M-β and MMM-β: DNP NMR data recorded at 9.4 T or 14.1 T using 3.2 mm rotors vs 9.4 T room temperature NMR data using 4 mm rotors which perhaps is the most commonly used solid state NMR configuration.

S3. Process for extraction the ^{29}Si-^{13}C dipolar couplings from the $^{29}Si\{^{13}C\}$ REDOR (Rotational Echo DOuble Resonance)[12] data

The vertical error bars in the $^{29}Si\{^{13}C\}$ REDOR curves (Figure 7, Figures S17-19) have been derived from both the signal-to-noise ratios of the experimentally obtained S_0 and S' spectra, and error analysis in the fit of all experimental ^{29}Si lineshapes. The signal-to-noise ratios were calculated from the Topspin 3.2 software while the error analysis was extracted from the DMFIT fitting software.[14]

For short dipolar evolution times ($\Delta S/S_0 < 0.25$), the REDOR curve is geometry-independent, and a first-order approximation can be applied, which gives the following analytical formula for the $^{29}Si\{^{13}C\}$ dephasing REDOR curves:[27]

$$\frac{\Delta S}{S_0} = \frac{16}{15}\tau^2 \sum_{i=1}^{n} D_i^2 \qquad \text{Equation S3}$$

where τ, and D_i are the recoupling times and the dipolar coupling constants (in Hz) of different ^{29}Si-^{13}C spin pairs, respectively. If assuming a ^{29}Si-^{13}C single spin pair model, the formula becomes:

$$\frac{\Delta S}{S_0} = \frac{16}{15}\tau^2 \times D^2 \qquad \text{Equation S4}$$

where D is the dipolar coupling constant (in Hz) of the ^{29}Si-^{13}C spin pair.

The relationship between the dipolar coupling constant (in Hz) and internuclear distance between ^{29}Si and ^{13}C spins ($r_{^{29}Si-^{13}C}$) is

$$D = - \frac{\mu_0}{8\pi^2} \frac{\hbar \gamma_{^{29}Si} \gamma_{^{13}C}}{r^3_{^{29}Si-^{13}C}} \qquad \text{Equation S5}$$

where $\gamma_{^{29}Si}$ and $\gamma_{^{13}C}$ are the gyromagnetic ratios of ^{29}Si and ^{13}C spins, respectively, $\hbar$ the reduced Planck's constant and μ_0 the vacuum permeability.

S4. Additional tables

Table S1. Enhancement factors $\Sigma^{\dagger}_{C\ CP}$ and $\Sigma^{\dagger}_{Si\ CP}$ calculation parameters for M-β with template.

	$\Sigma^{\dagger}_{C\ CP}$			$\Sigma^{\dagger}_{Si\ CP}$		
Matrix	20 mM TEKPol[5] in TCE[a]	TCE	None	20 mM TEKPol in TCE	TCE	None
Field / T	9.4	9.4	9.4	9.4	9.4	9.4
ε	14	-	-	14	-	-
Λ / Hz	210[b]	-	100[b]	420	-	360
T / K	110[c]	-	298	110[c]	-	298
(S/N) / a.u.	208	37	41	129	28	38
m / mg	23	24	89	23	24	89
NS	32	64	1024	16	64	512
τ_{DNP} / s	2.1	-	-	2.1	-	-
T_1 / s	-	-	0.17[d]	-	-	0.17[d]
θ	0.6	-	-	0.7	-	-
$\Sigma^{\dagger}$	25	-	-	34	-	-

[a] "TCE" refers to 1,1,2,2-tetrachloroethane. [b] Signal of CH_2 group of $(CH_3CH_2)_4NOH$. [c] Calculated through T_1 of ^{79}Br in KBr added to rotor.[7] [d] A longer recycle delay of 3 s (instead of 1.3 × T_1) was used to record the CP experiments as to ensure an adequate duty cycle for the probe.

Table S2. Enhancement factors $\Sigma^{\dagger}_{C\ CP}$ and $\Sigma^{\dagger}_{Si\ CP}$ calculation parameters for MMM-β with template.

	$\Sigma^{\dagger}_{C\ CP}$			$\Sigma^{\dagger}_{Si\ CP}$		
Matrix	20 mM TEKPol in TCE	TCE	None	20 mM TEKPol in TCE	TCE	None
Field / T	9.4	9.4	9.4	9.4	9.4	9.4
ε	54	-	-	72	-	-
Λ / Hz	210[a]	-	70[a]	500	-	320
T / K	110[b]	-	298	110[b]	-	298
(S/N) / a.u.	364	18	35	670	19	45
m / mg	12	13	60	12	13	60
NS	64	128	512	64	64	256
τ_{DNP} / s	2.0	-	-	2.0	-	-
T_1 / s	-	-	0.92[c]	-	-	0.92[c]
θ	0.6	-	-	0.5	-	-
$\Sigma^{\dagger}$	53	-	-	97	-	-

[a] Signal of CH_2 group of $(CH_3CH_2)_4NOH$. [b] Calculated through T_1 of ^{79}Br in KBr added to rotor.[7]
[c] A longer recycle delay of 3 s (instead of 1.3 × T_1) was used to record the CP experiments as to ensure an adequate duty cycle for the probe.

Table S3. Concentration of electron spins in the DNP samples.

Sample	Electron spin concentration measured by EPR spin counting (mmol/g)[a]	Electron spin concentration calculated from preparation procedure (mmol/g)[b]
M-β with template	$(2.7 \pm 0.8) \times 10^{-2}$	$(2.2 \pm 1.7) \times 10^{-2}$
Activated M-β	$(1.1 \pm 0.3) \times 10^{-2}$	$(2.4 \pm 1.8) \times 10^{-2}$
MMM-β with template	$(7.4 \pm 2.2) \times 10^{-2}$	$(3.3 \pm 2.5) \times 10^{-2}$
Activated MMM-β	$(7.3 \pm 2.2) \times 10^{-2}$	$(3.3 \pm 2.5) \times 10^{-2}$

[a] EPR spin counting measurements were performed on samples after DNP experiments. The error on the spin counting measurements was estimated to be about ±30%.[15,16] [b] The electron spin concentrations were obtained based on the amount of zeolite and radical solution added. The error was estimated to be about ±75%, considering the evaporation of TCE solvent, error in radical concentration in solution, error in sample mass, etc.[17]

Table S4. Enhancement factors $\Sigma^{\dagger}_{C\ CP}$ and $\Sigma^{\dagger}_{Si\ CP}$ calculation parameters for activated M-β.

	$\Sigma^{\dagger}_{C\ CP}$					$\Sigma^{\dagger}_{Si\ CP}$		
Matrix	20 mM TEKPol in TCE		TCE		None	20 mM TEKPol in TCE	TCE	None
Field / T	9.4	14.1	9.4	14.1	9.4	9.4	9.4	9.4
ε	10	4	-	-	-	9	-	-
Λ / Hz	490[a]	900[a]	-	-	365[a]	520	-	400
T / K	110[b]	125[b]	-	-	298	110[b]	-	298
(S/N) / a.u.	851	274	192	217	242	273	50	108
m / mg	22	14	20	15	75	17	15	75
NS	32	32	128	160	1024	16	32	5656
τ_{DNP} / s	10.2	13	-	-	-	9.7	-	-
T_1 / s	-	-	-	-	0.74[c]	-	-	0.74[c]
θ	0.8	0.8	-	-	-	0.8	-	-
$\Sigma^{\dagger}$	30	6[d]	-	-	-	96	-	-

[a] Signal of methanol. [b] Calculated through T_1 of ^{79}Br in KBr added to rotor.[7] [c] A longer recycle delay of 2 s or 3 s (instead of 1.3 × T_1) was used to record the CP experiments as to ensure an adequate duty cycle for the probe. [d] Compared to the 9.4 T room temperature spectrum.

Table S5. Enhancement factors $\Sigma^{\dagger}_{C\,CP}$ and $\Sigma^{\dagger}_{Si\,CP}$ calculation parameters for activated MMM-β.

	$\Sigma^{\dagger}_{C\,CP}$						$\Sigma^{\dagger}_{Si\,CP}$		
Matrix	20 mM TEKPol in TCE			TCE		None	20 mM TEKPol in TCE	TCE	None
Field / T	9.4[a]	9.4	14.1	9.4[a]	14.1	9.4	9.4	9.4	9.4
ε	34	40	9	-	-	-	45	-	-
Λ / Hz	2900[b]	2800[b]	4500[b]	-	-	3100[b]	500	-	570
T / K	110[c]	110[c]	125[c]	-	-	298	110[c]	-	298
(S/N) / a.u.	325	168	159	15	58	89	854	41	20
m / mg	13	11	13	15	14	53	13	15	53
NS	128	32	64	128	256	20480	64	128	1024
τ_{DNP} / s	4.2	2.9	4.3	-	-	-	4.2	-	-
T_1 / s	-	-	-	-	-	0.31[d]	-	-	0.31[d]
θ	0.7	0.7	0.7	-	-	-	0.8	-	-
$\Sigma^{\dagger}$	111	161	38[e]	-	-	-	462	-	-

[a] Used to calculate θ. [b] Signal of aromatic rings. [c] Calculated through T_1 of ^{79}Br in KBr added to rotor.[7] [d] A longer recycle delay of 2 s or 3 s (instead of 1.3 × T_1) was used to record the CP experiments as to ensure an adequate duty cycle for the probe. [e] Compared to the 9.4 T room temperature spectrum.

Table S6. Framework Si/Al ratios in H-type M-β and MMM-β zeolites determined from ^{29}Si MAS NMR spectra (Figure S20 for MMM-β).

Zeolite	Framework Si/Al
M-β	24[2]
MMM-β	34

S5. Additional figures

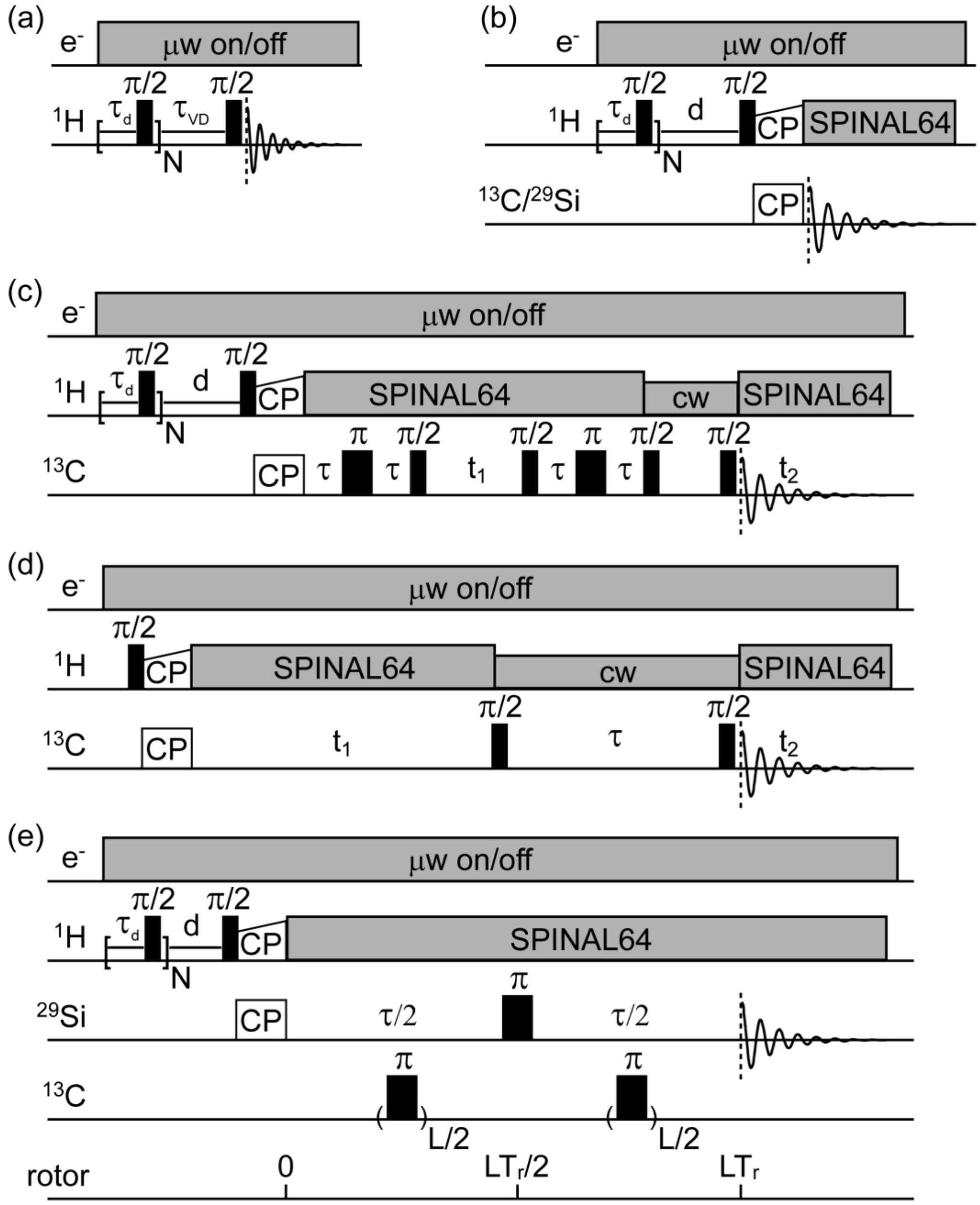

Figure S1. (a) ^{1}H saturation recovery pulse sequence to measure DNP polarization build-up time ($\tau_{DNP}(^{1}H)$) with μw on/off and ^{1}H longitudinal relaxation time constant (T_1) at room temperature. τ_{vd} is the variable delay list. (b) Pre-saturated CP pulse sequence. (c) Pre-saturated ^{13}C-^{13}C CP z-filtered refocused INADEQUATE pulse sequence. τ is synchronized to be an integer number of rotor periods.[8] (d) ^{13}C-^{13}C CP PDSD DARR pulse sequence with τ representing the mixing time.[9–11] (e) $^{29}Si\{^{13}C\}$ CP REDOR pulse sequence.[12] $T_r = 1/\nu_r$ is the rotor period, L is number of rotor periods and τ is the total recoupling time equaling to $L{*}T_r$. In all the sequences, d is the recycle delay which is equal to $1.3 \times \tau_{DNP}(^{1}H)$. N = 100 is the number of ^{1}H saturation pulses with 1 ms delay τ_d between pulses. t_1 and t_2 correspond to indirect and direct dimension acquisition time, respectively. “CW” refers to continuous wave.

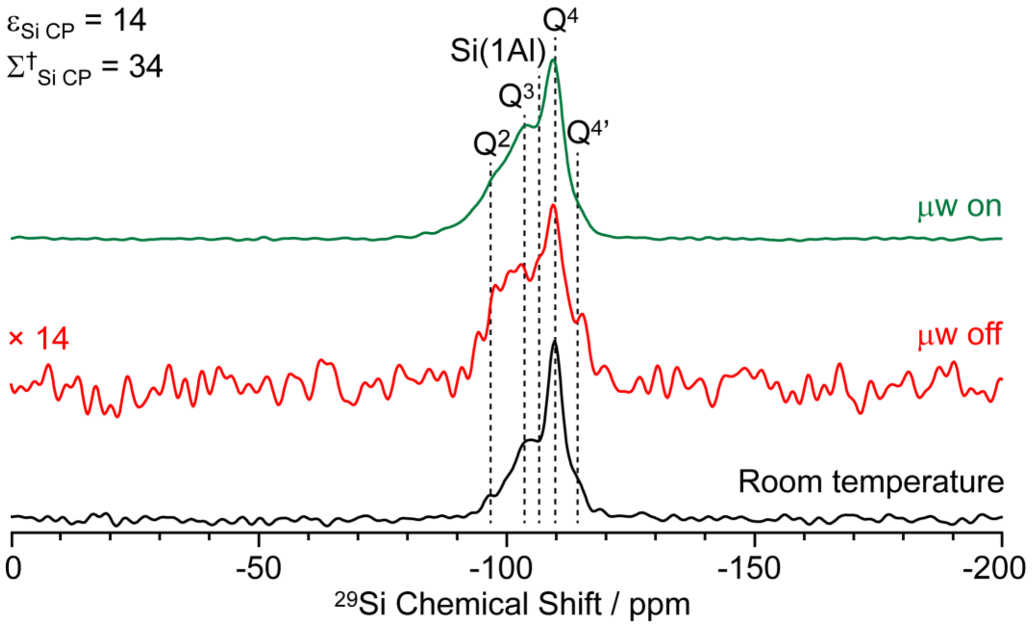

Figure S2. ^{29}Si CP MAS DNP spectra of M-β with template with microwave irradiation (μw on) (green) and without (μw off) (red) recorded at 110 K. The sample was impregnated with 20 mM TEKPol in TCE. The black spectrum shows the ^{29}Si CP MAS spectrum at room temperature (without adding the radical solution). The experimental times for the μw on/off and room temperature spectra are 45 seconds and 26 minutes, respectively. All spectra were recorded at 9.4 T and with a MAS rate of 8 kHz.

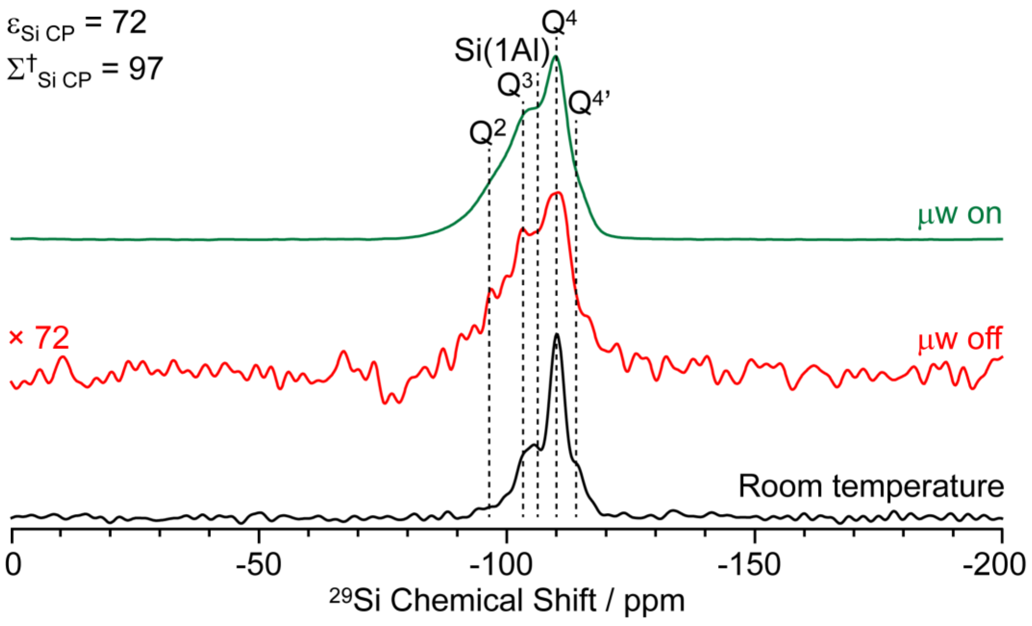

Figure S3. ^{29}Si CP MAS DNP spectra of MMM-β with template with microwave irradiation (μw on) (green) and without (μw off) (red) recorded at 110 K. The sample was impregnated with 20 mM TEKPol in TCE. The black spectrum the ^{29}Si CP MAS spectrum at room temperature (without adding the radical solution). The experimental times for the μw on/off and room temperature spectra are 166 seconds and 13 minutes, respectively. All spectra were recorded at 9.4 T and with a MAS rate of 8 kHz.

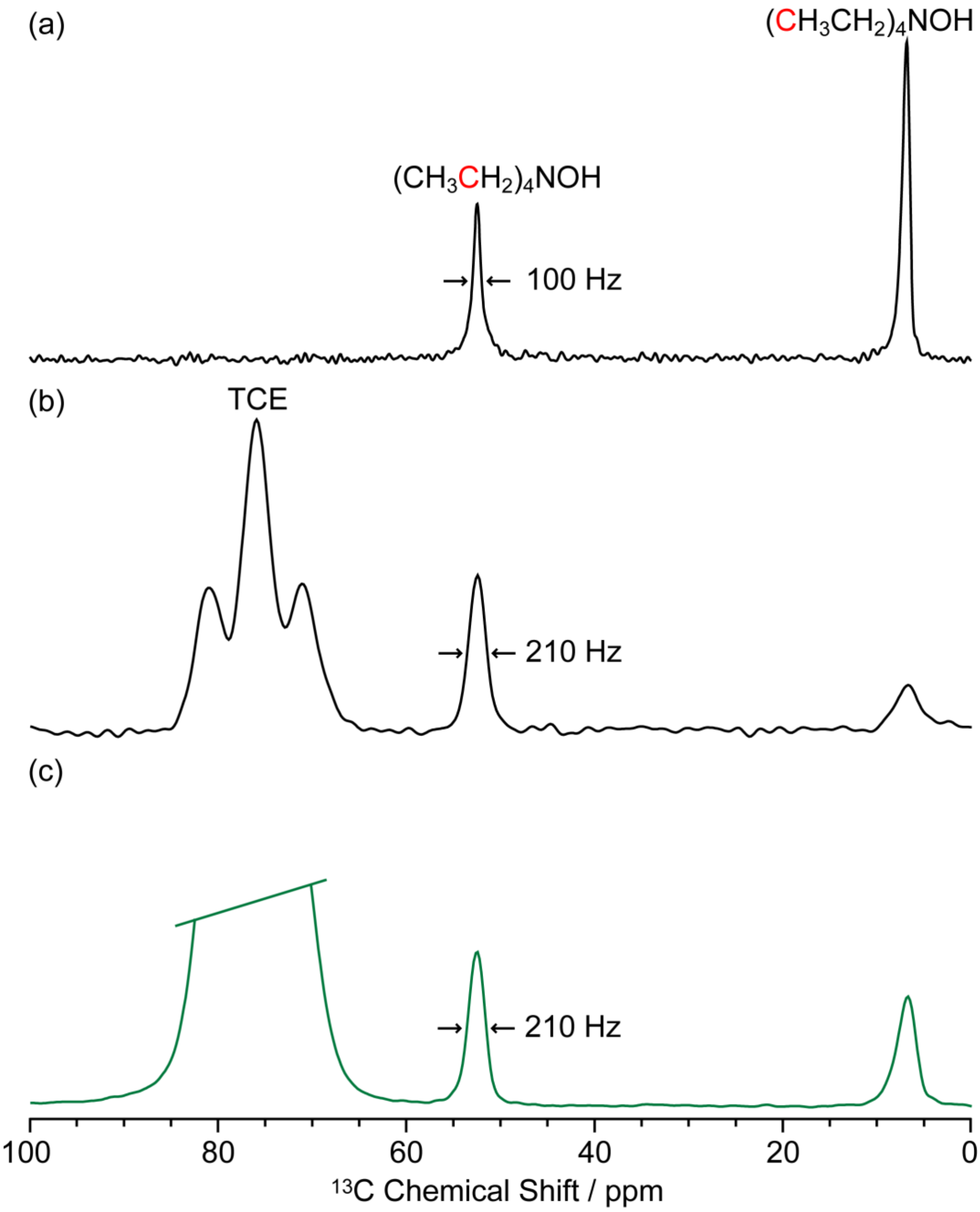

Figure S4. ^{13}C CP MAS spectra of M-β with template. (a) Spectrum of the neat zeolite at room temperature. (b) Spectrum of the zeolite impregnated with TCE at 110 K. (c) DNP μw on spectrum of the zeolite impregnated with 20 mM TEKPol in TCE at 110 K. All spectra were recorded at 9.4 T and with a MAS rate of 12.5 kHz.

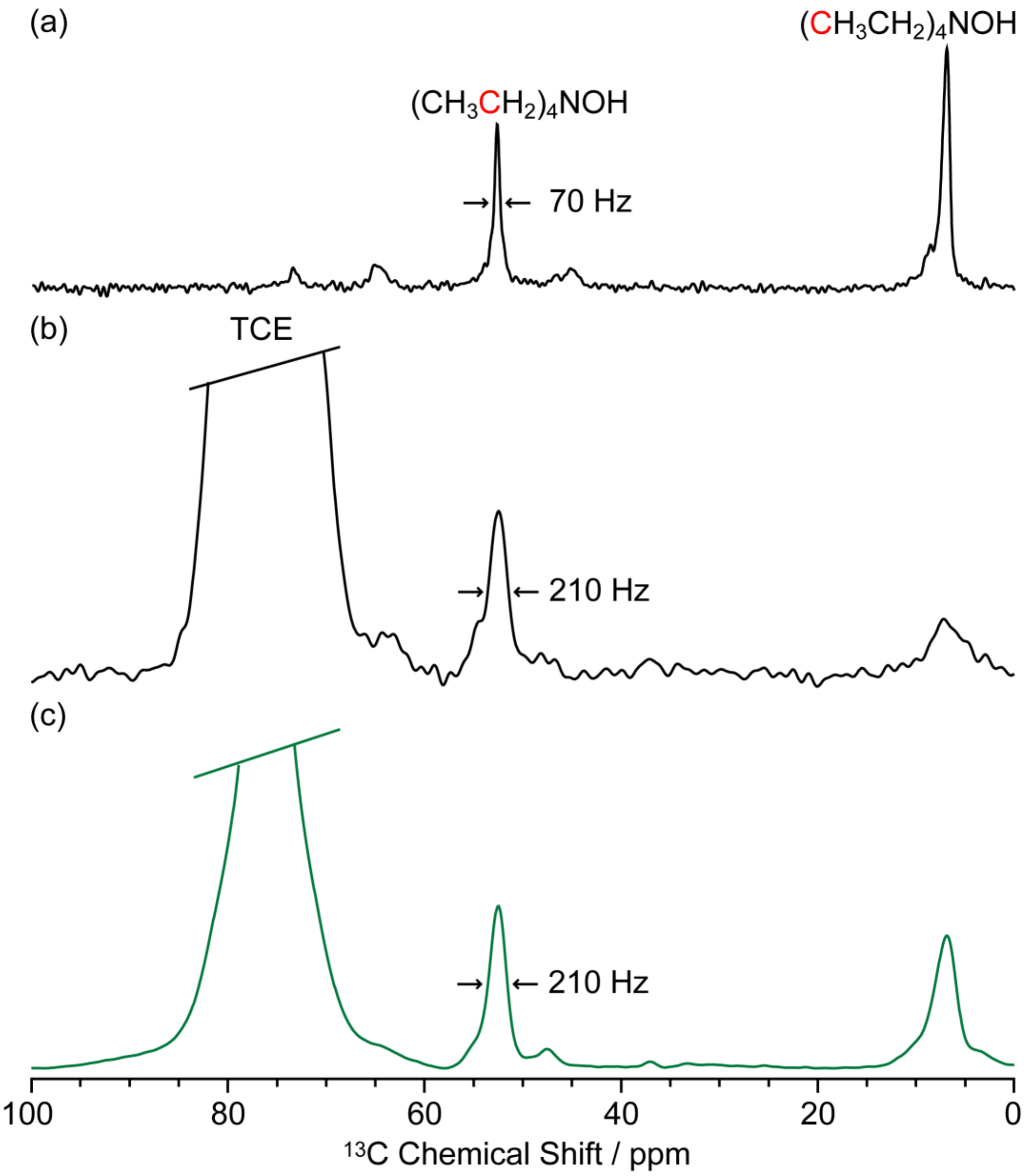

Figure S5. ^{13}C CP MAS spectra of MMM-β with template. (a) Spectrum of the neat zeolite at room temperature. (b) Spectrum of the zeolite impregnated with TCE at 110 K. (c) DNP μw on spectrum of the zeolite impregnated with 20 mM TEKPol in TCE at 110 K. All spectra were recorded at 9.4 T and with a MAS rate of 12.5 kHz.

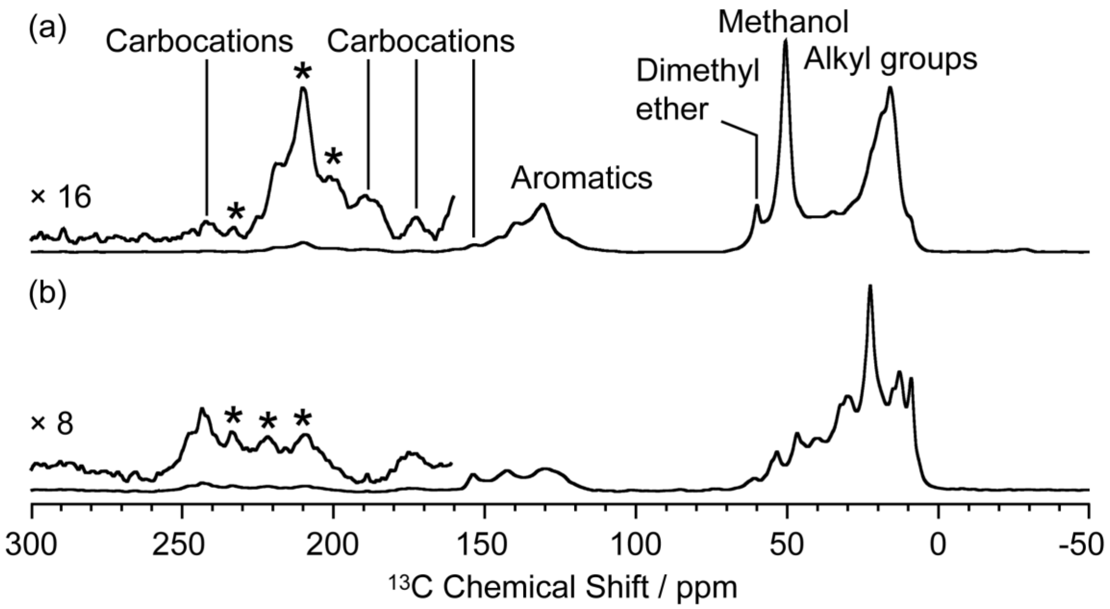

Figure S6. Room temperature ^{13}C CP MAS spectra of (a) MMM-β activated with $^{13}CH_3OH$ and (b) MMM-β activated with $^{13}C_2H_4$. Data were recorded at 14.1 T and with a MAS rate of 12 kHz. Asterisks (*) denote spinning sidebands.

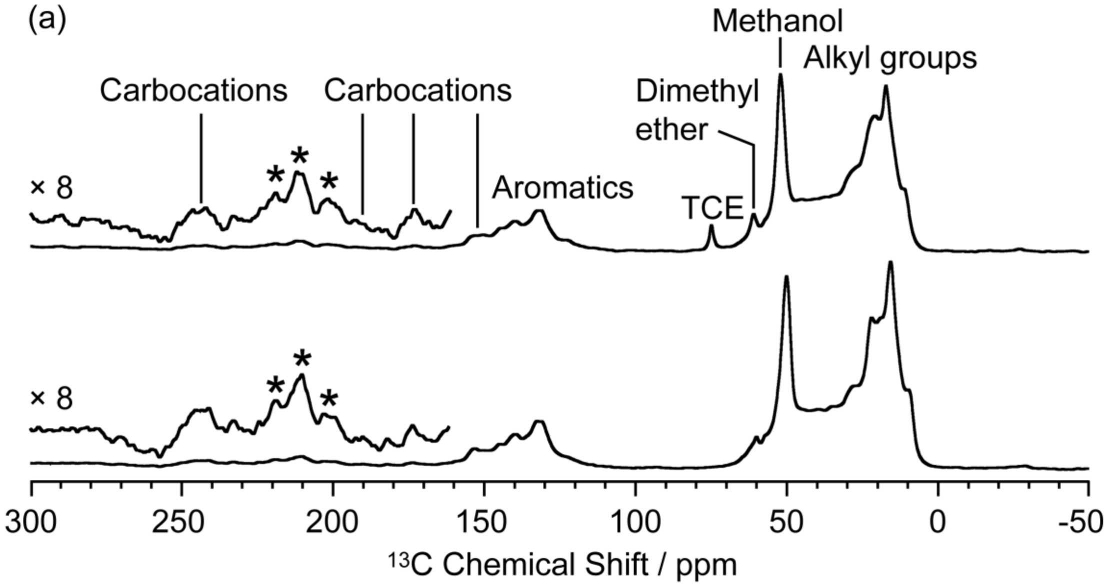

Figure S7. Room temperature ^{13}C CP MAS spectra of (a) activated M-β and (b) activated MMM-β. The upper and lower spectra were obtained on zeolites impregnated with TCE and neat, respectively. Data were recorded at 14.1 T and with a MAS rate of 12 kHz. Asterisks (*) denote spinning sidebands.

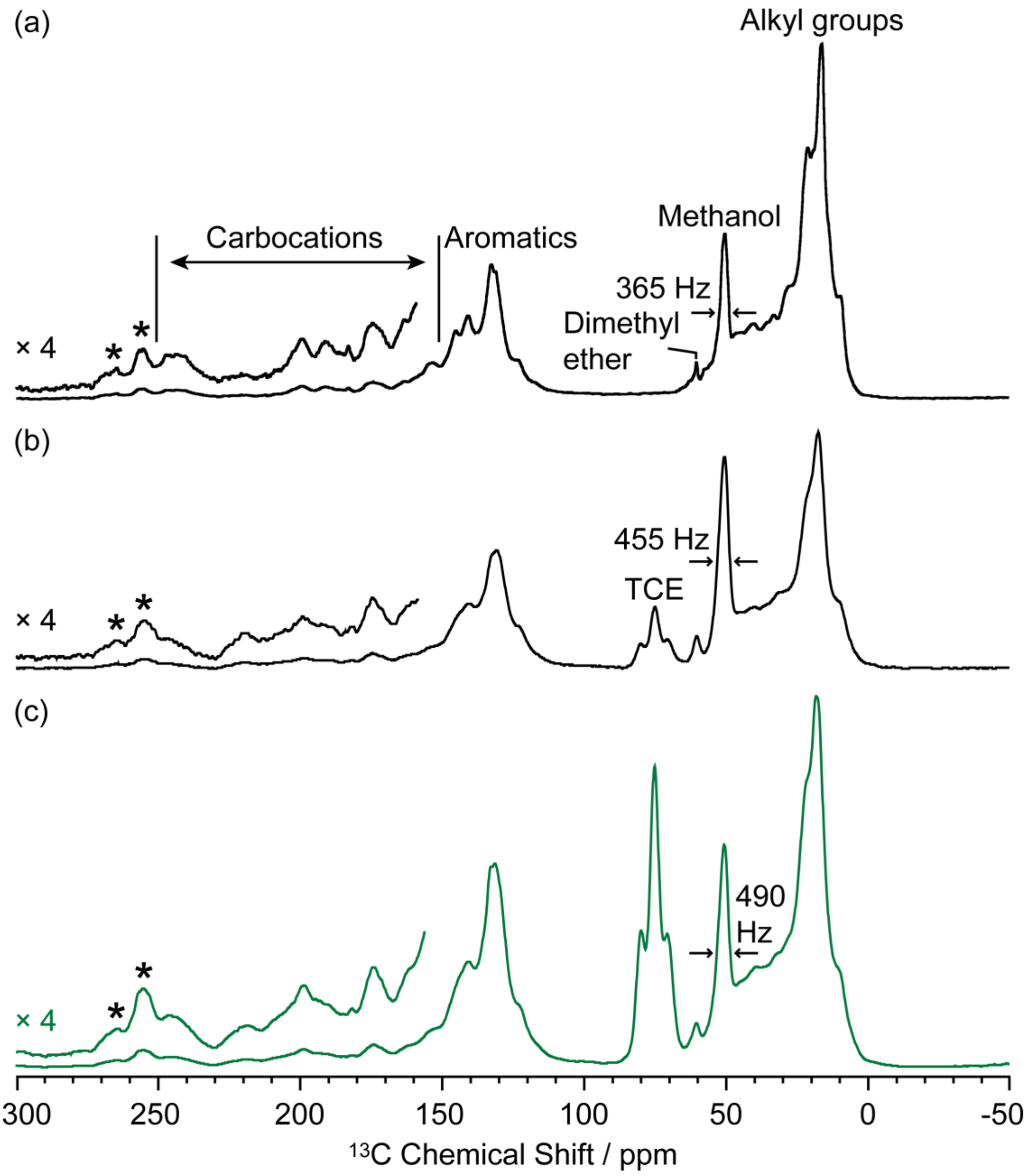

Figure S8. ^{13}C CP MAS spectra of activated M-β. (a) Spectrum of the neat activated zeolite at room temperature. (b) Spectrum of the activated zeolite impregnated with TCE at 110 K. (c) DNP μw on spectrum of the activated zeolite impregnated with 20 mM TEKPol in TCE at 110 K. All spectra were recorded at 9.4 T and with a MAS rate of 12.5 kHz. Asterisks (*) denote spinning sidebands.

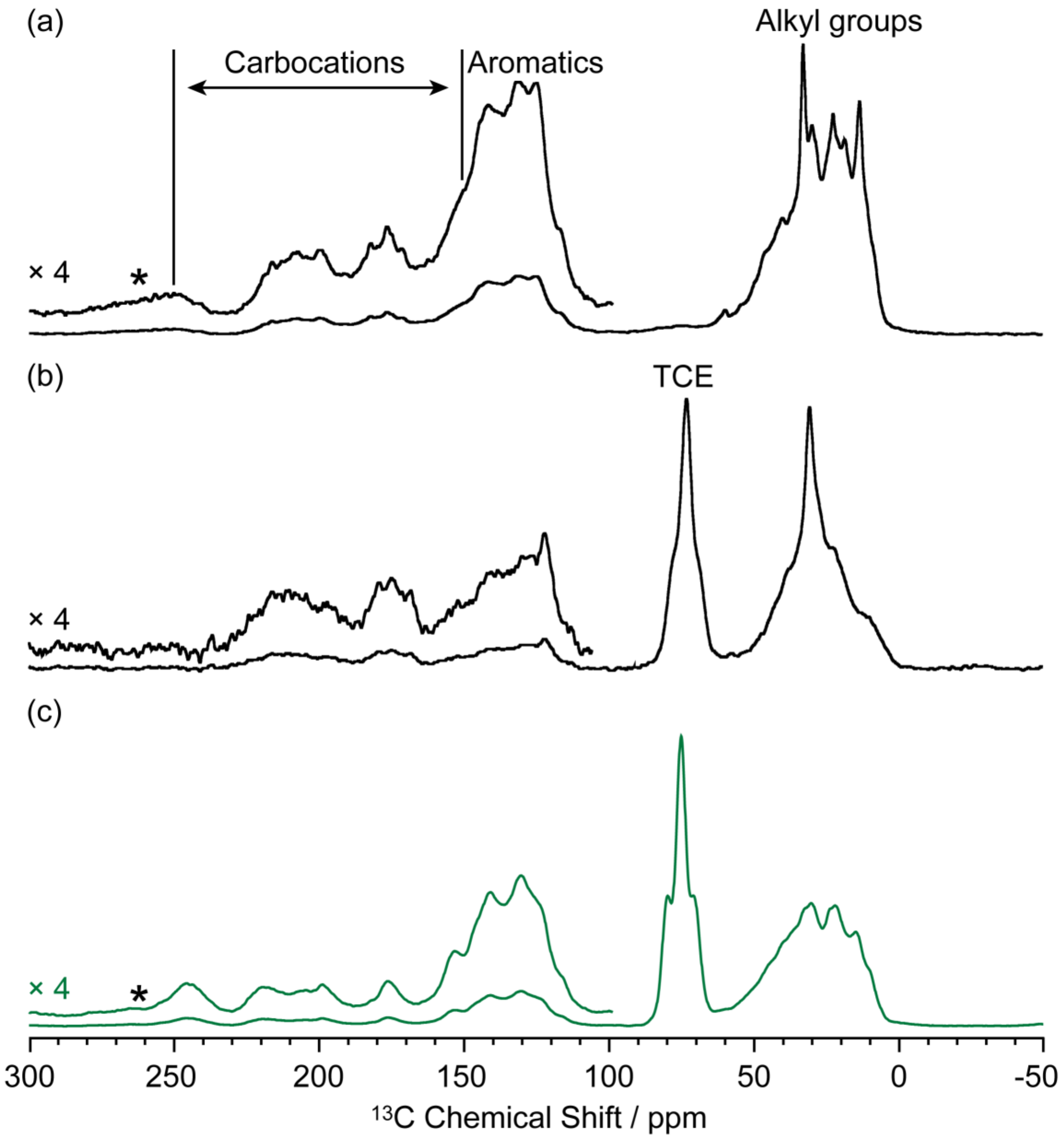

Figure S9. ^{13}C CP MAS spectra of activated MMM-β. (a) Spectrum of the neat activated zeolite at room temperature. (b) Spectrum of the activated zeolite impregnated with TCE at 110 K. (c) DNP μw on spectrum of the activated zeolite impregnated with 20 mM TEKPol in TCE at 110 K. All spectra were recorded at 9.4 T and with a MAS rate of 12.5 kHz. Asterisks (*) denote spinning sidebands.

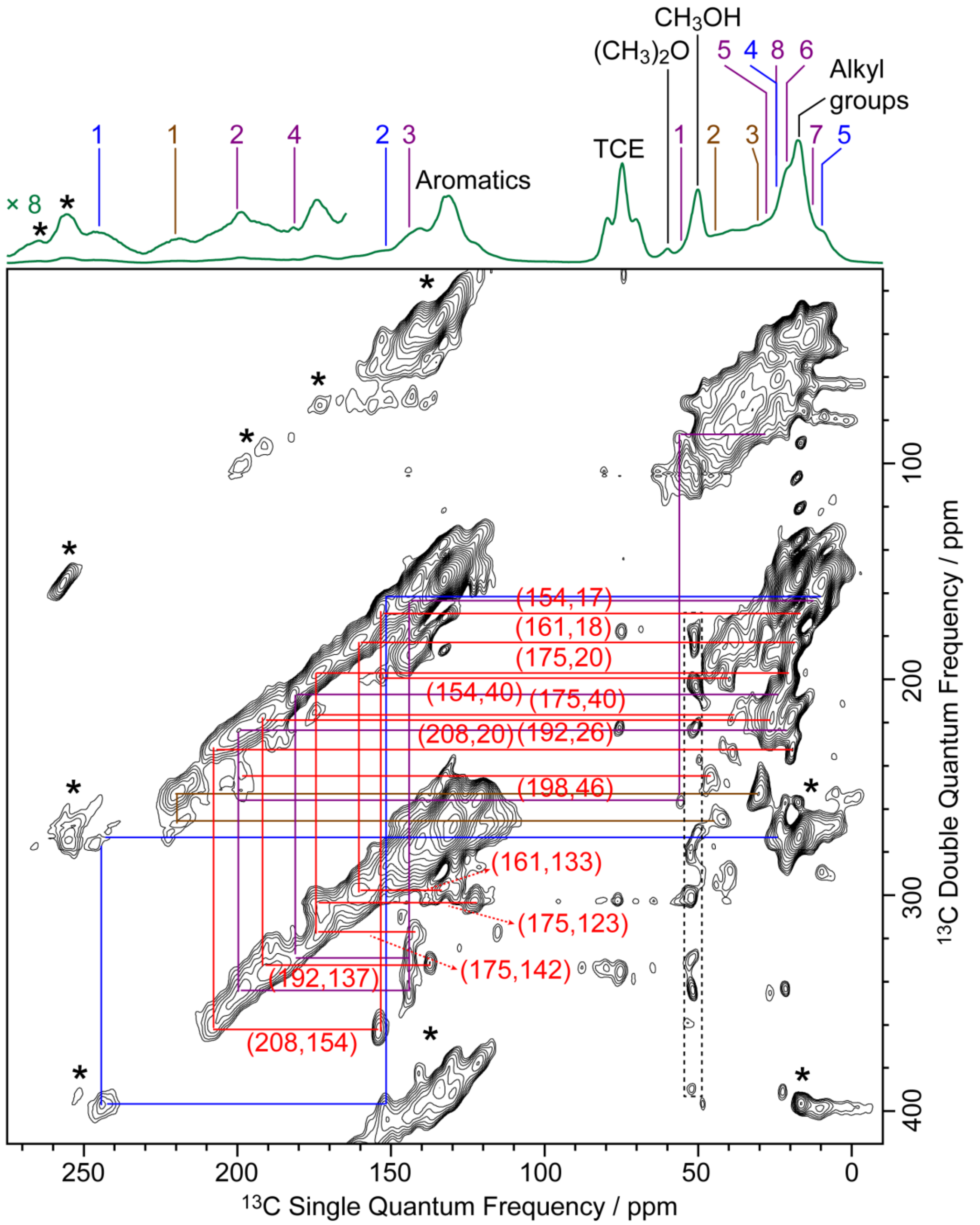

Figure S10. DNP enhanced 2D ^{13}C-^{13}C refocused INADEQUATE spectrum of activated M-β. Data was recorded at B_0 = 9.4 T and with a MAS frequency of ν_r = 12.5 kHz. The correlations and spectral assignments are coded with the same colors as their corresponding carbocations in Figure 2. Signals in the black dashed box correspond to t_1 noise. Correlations corresponding to naphthalenium ions (**IV**) are shown with the ^{13}C chemical shifts in the Single Quantum dimension of the two correlated carbon atoms given in the parenthesis. Correlations for cation **II** are C1(**II**) (56 ppm) – C2(**II**) (199 ppm), C1(**II**) (56 ppm) – C5(**II**) (28 ppm), C2(**II**) (199 ppm) – C3(**II**) (144 ppm), C2(**II**) (199 ppm) – C6(**II**) (22 ppm), C3(**II**) (144 ppm) – C4(**II**) (182 ppm), C3(**II**) (144 ppm) – C7(**II**) (13 ppm), C4(**II**) (182 ppm) – C8(**II**) (24 ppm), while for cation **III** are C1(**III**) (220 ppm) – C2(**III**) (45 ppm) and C1(**III**) (220 ppm) – C3(**III**) (30 ppm). Asterisks (*) denote spinning sidebands.

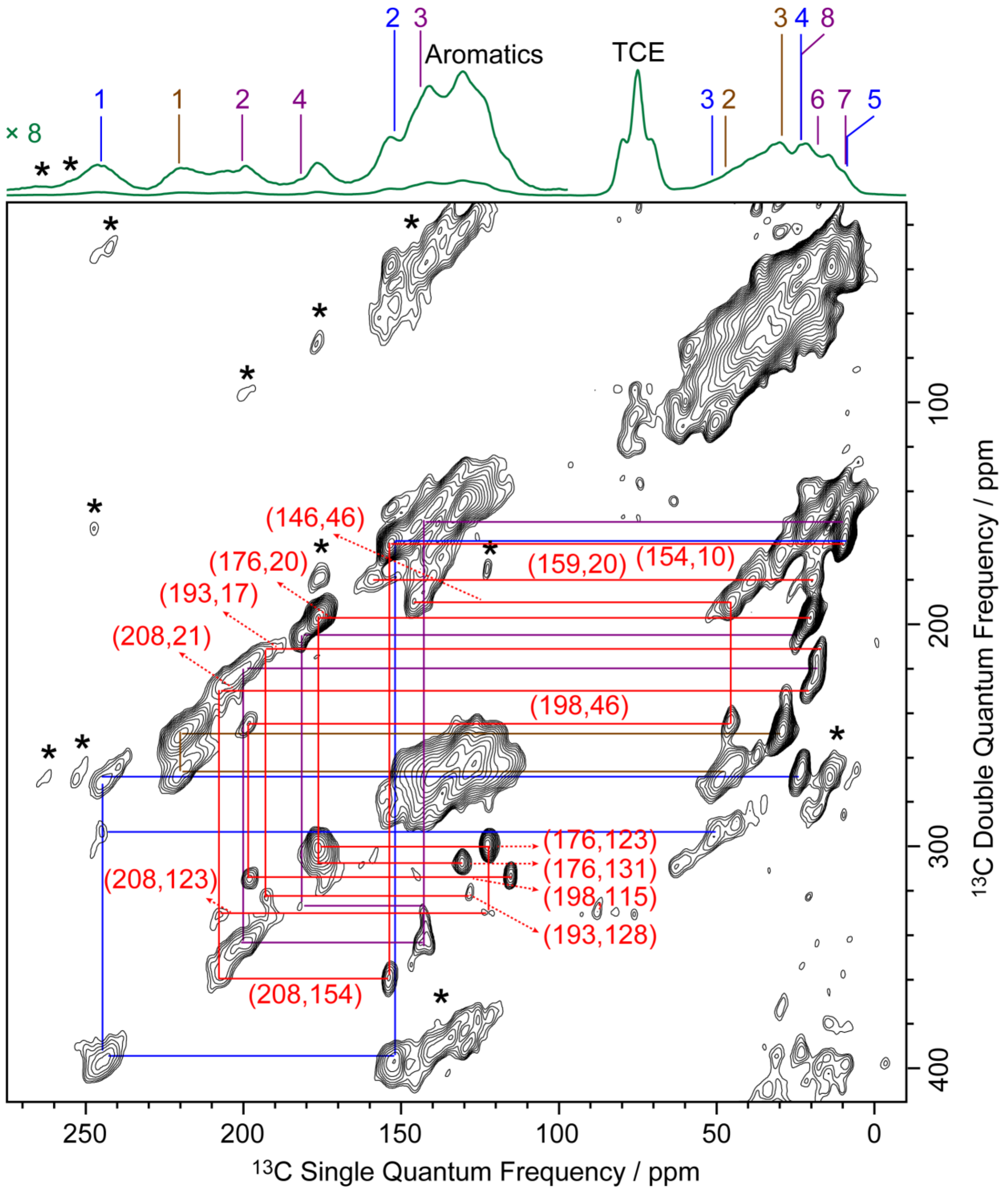

Figure S11. DNP enhanced 2D ^{13}C-^{13}C refocused INADEQUATE spectrum of activated MMM-β. Data was recorded at B_0 = 9.4 T and with a MAS frequency of ν_r = 12.5 kHz. The correlations and spectral assignments are coded with the same colors as their corresponding carbocations in Figure 2. Correlations corresponding to naphthalenium ions (**IV**) are shown with the ^{13}C chemical shifts in the Single Quantum dimension of the two correlated carbon atoms given in the parenthesis. Correlations for cation **II** are C2(**II**) (199 ppm) – C3(**II**) (144 ppm), C2(**II**) (199 ppm) – C6(**II**) (19 ppm), C3(**II**) (144 ppm) – C4(**II**) (182 ppm), C3(**II**) (144 ppm) – C7(**II**) (10 ppm), C4(**II**) (182 ppm) – C8(**II**) (24 ppm), while for cation **III** are C1(**III**) (220 ppm) – C2(**III**) (48 ppm) and C1(**III**) (220 ppm) – C3(**III**) (30 ppm). Asterisks (*) denote spinning sidebands.

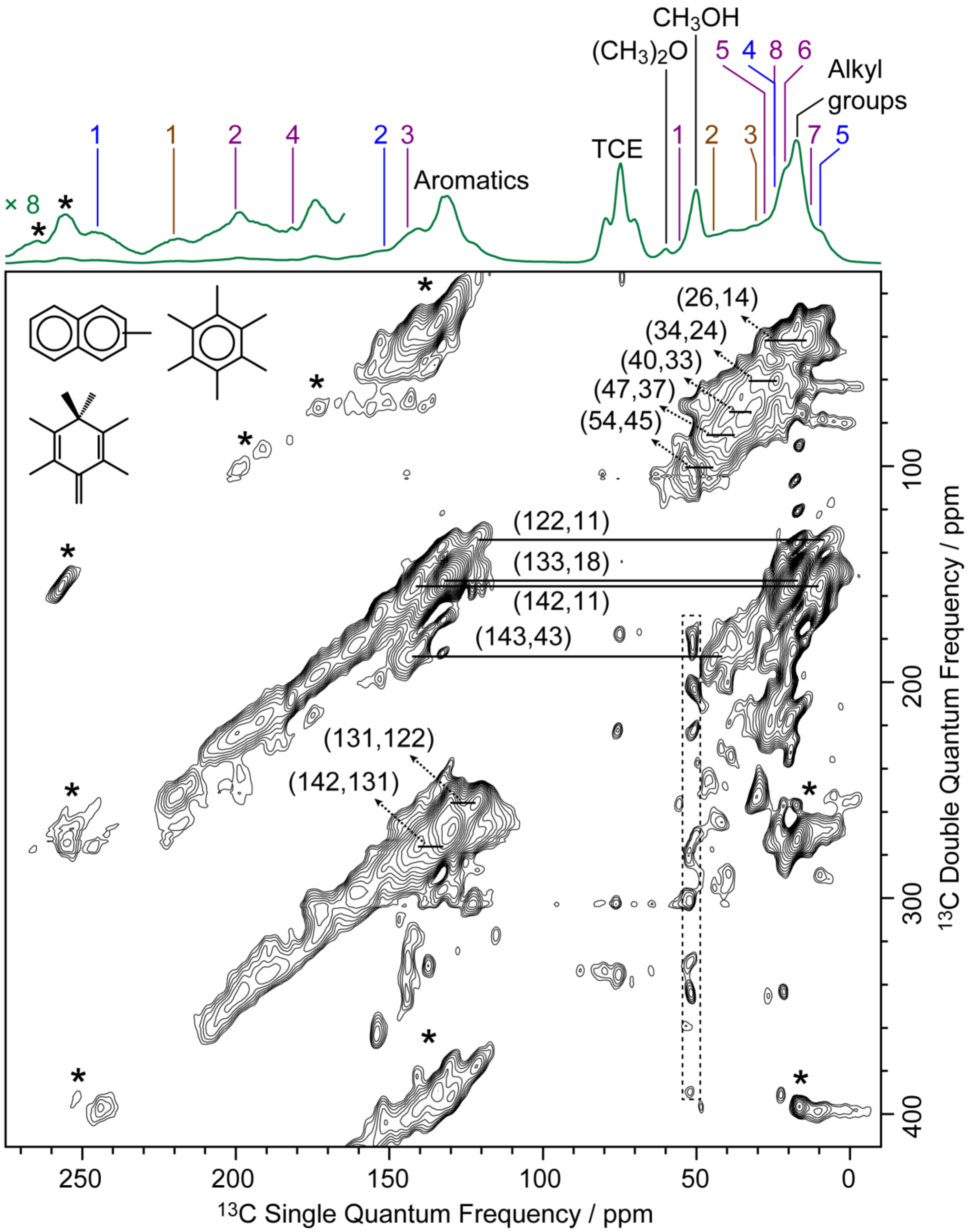

Figure S12. DNP enhanced 2D ^{13}C-^{13}C refocused INADEQUATE spectrum of activated M-β. Data was recorded at B_0 = 9.4 T and a MAS frequency of v_r = 12.5 kHz. Signals in the black dashed box correspond to t_1 noise. Correlations corresponding to the neutral species such as aromatics and alkanes[2] are shown with the ^{13}C chemical shifts in the Single Quantum dimension of the two correlated carbon atoms given in the parenthesis. Structures of such possible compounds are shown in the figure and include methylnaphthalenes,[28,29] hexamethylbenzene,[28,30] hexamethylmethylenecyclohexadiene.[31,32] Asterisks (*) denote spinning sidebands.

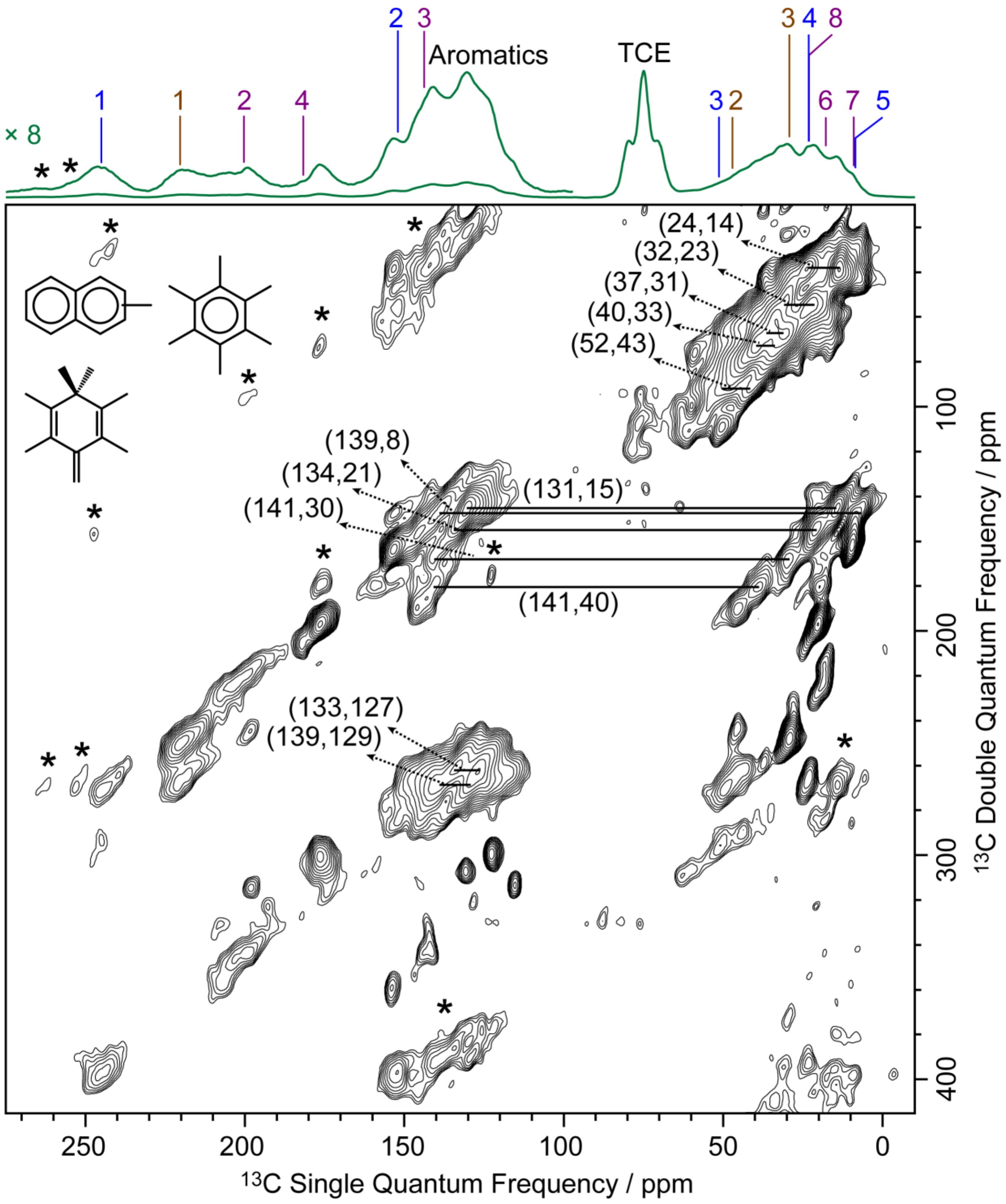

Figure S13. DNP enhanced 2D ^{13}C-^{13}C refocused INADEQUATE spectrum of activated MMM-β. Data was recorded at B_0 = 9.4 T and a MAS frequency of ν_r = 12.5 kHz. Correlations corresponding to the neutral species such as aromatics and alkanes[2] are shown with the ^{13}C chemical shifts in the Single Quantum dimension of the two correlated carbon atoms given in the parenthesis. Structures of such possible compounds are shown in the figure and include methylnaphthalenes,[28,29] hexamethylbenzene,[28,30] hexamethylmethylenecyclohexadiene.[31,32] Asterisks (*) denote spinning sidebands.

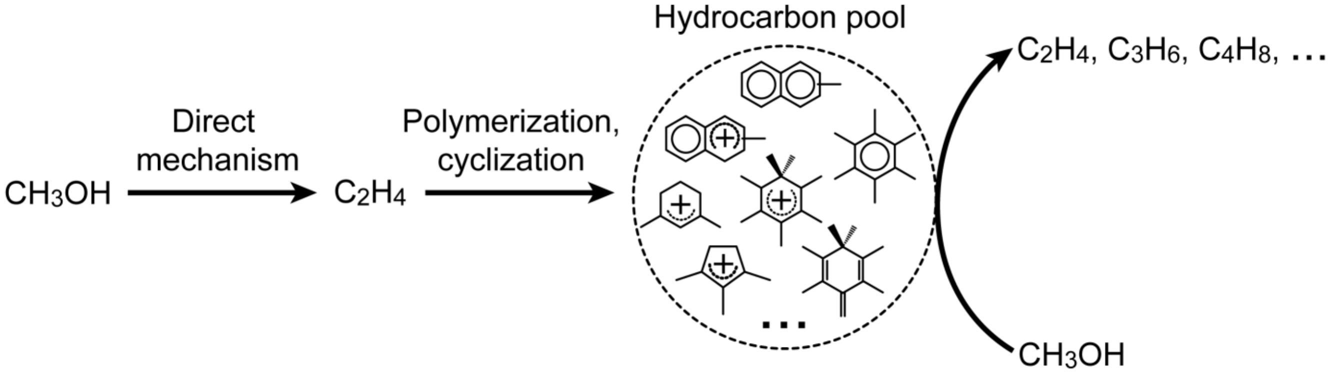

Figure S14. Reaction route for the formation of hydrocarbon pool species.[33–35]

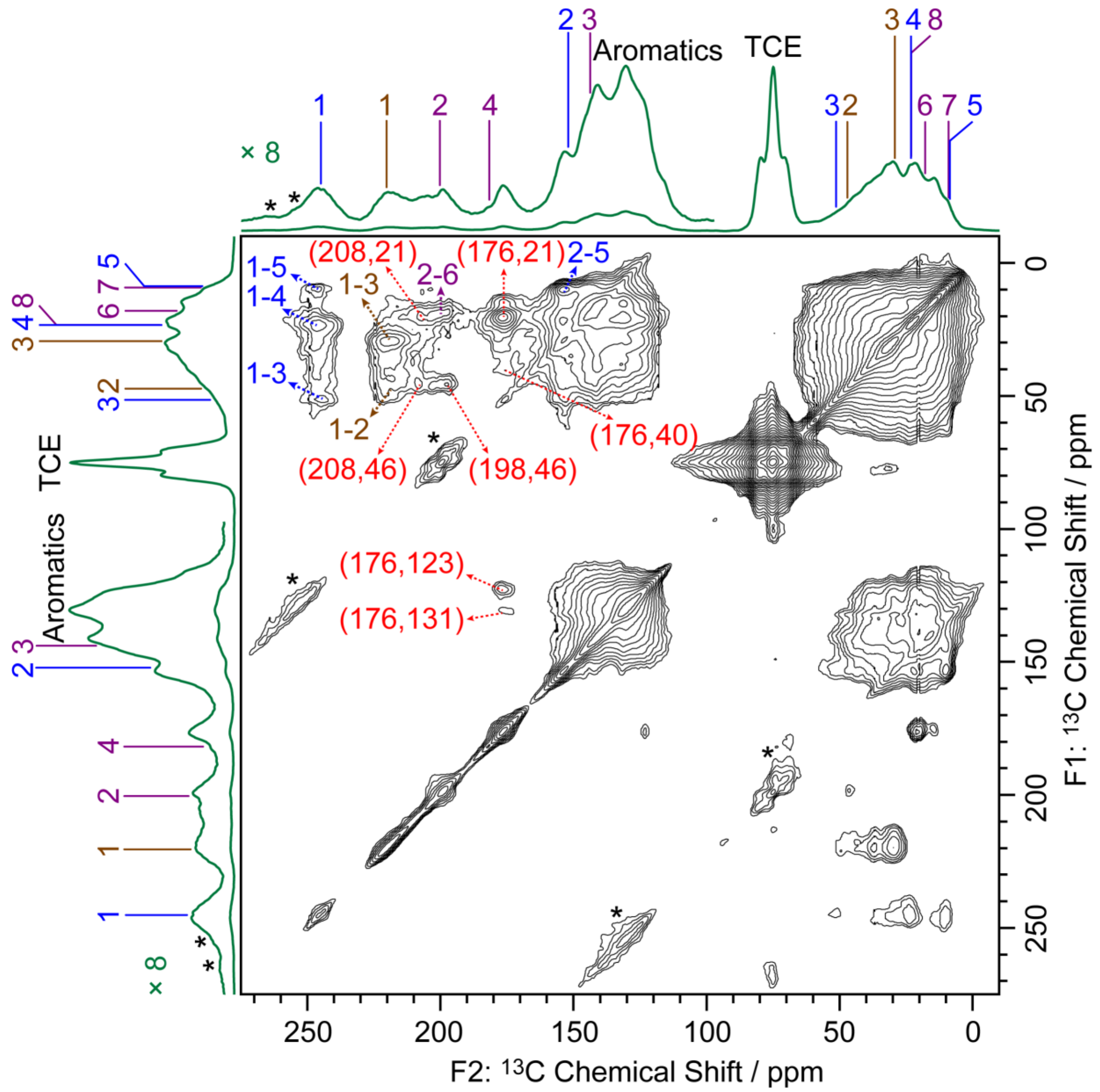

Figure S15. DNP enhanced 2D ^{13}C-^{13}C PDSD DARR spectrum of activated MMM-β. Data was recorded at B_0 = 9.4 T and with a MAS frequency of ν_r = 12.5 kHz. The correlations and spectral assignments are coded with the same colors as their corresponding carbocations in Figure 2. Correlations corresponding to naphthalenium ions (**IV**) are shown with the ^{13}C chemical shifts of the two correlated carbon atoms given in the parenthesis. Asterisks (*) denote spinning sidebands.

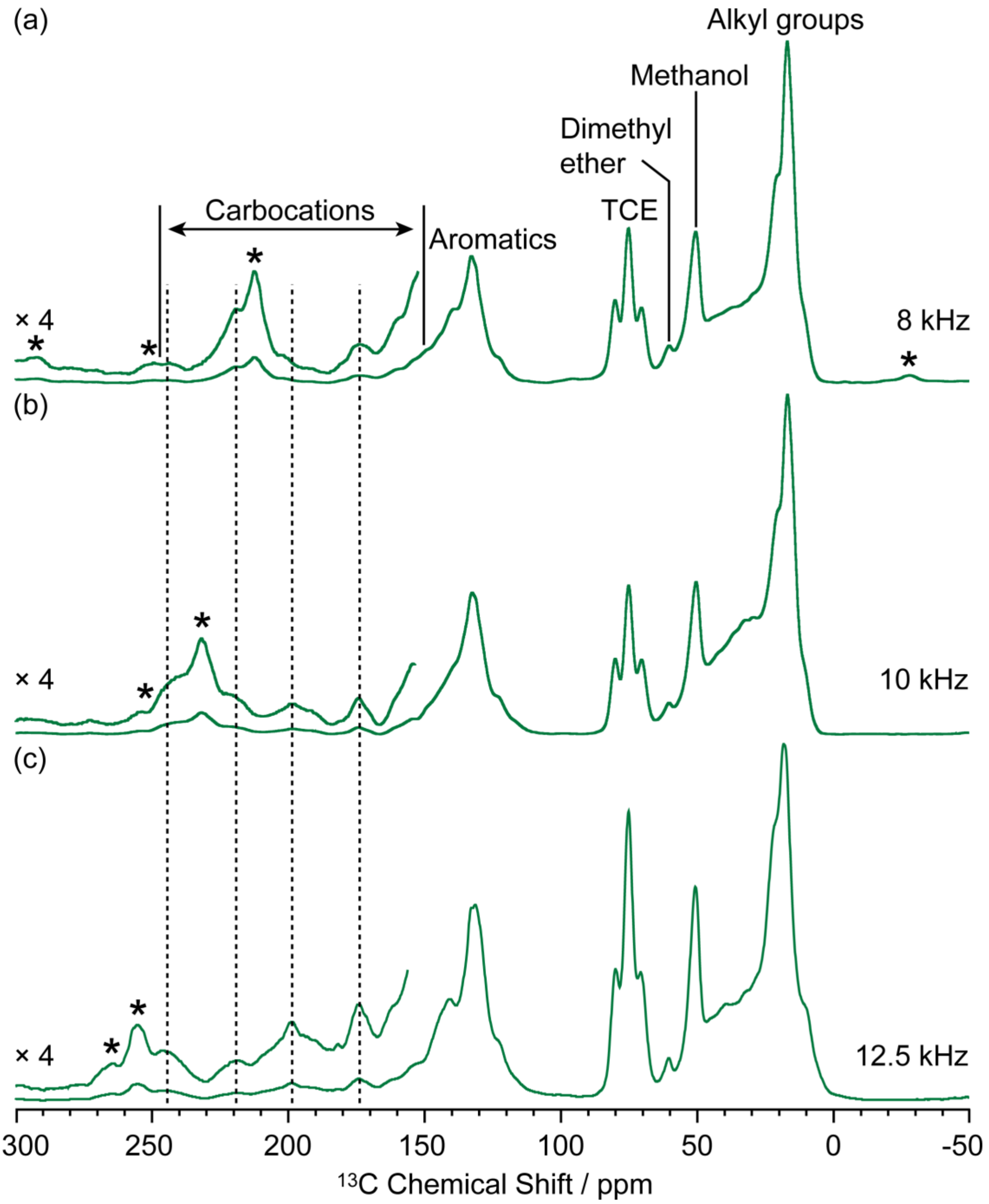

Figure S16. ^{13}C CP MAS DNP spectra of activated M-β. Data were recorded at 9.4 T and with a MAS rate of (a) 8 kHz, (b) 10 kHz and (c) 12.5 kHz. Asterisks (*) denote spinning sidebands.

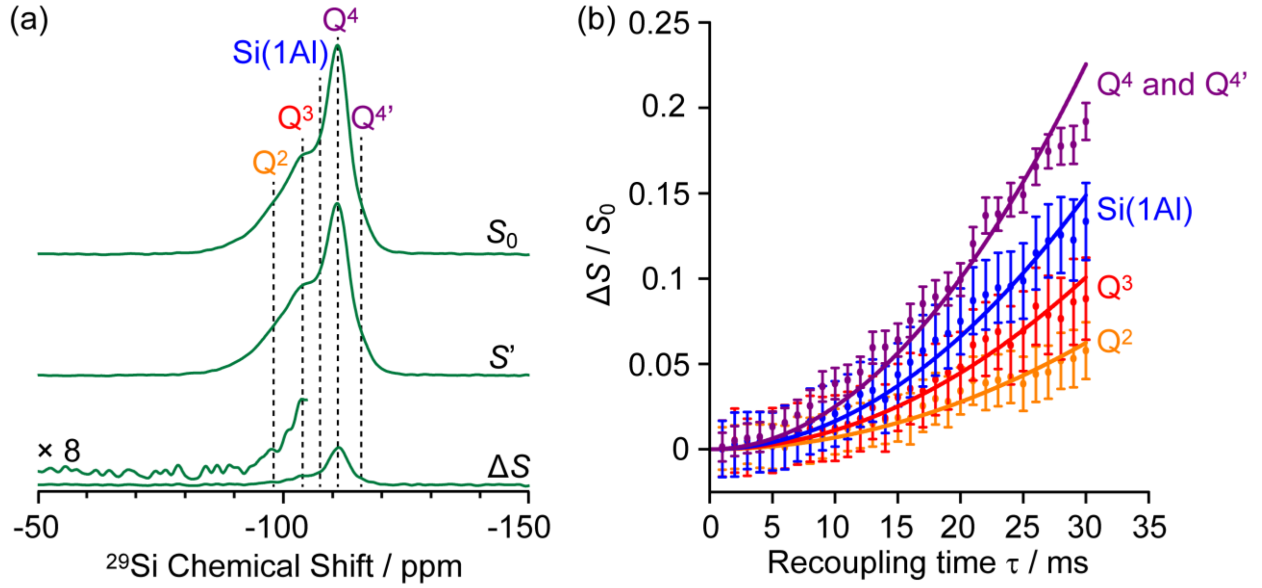

Figure S17. (a) DNP enhanced ^{29}Si CP spin echo spectrum (S_0) and ^{29}Si{^{13}C} REDOR spectrum (S') with the reintroduction of dipolar couplings at a recoupling time of 30 ms. ΔS is the difference spectrum S_0 - S'. Spectra were recorded at 9.4 T on activated MMM-β. (b) ^{29}Si{^{13}C} REDOR fraction $\Delta S/S_0$ as a function of the recoupling times up to 30 ms. The experimental time is ≈ 11 hours. The solid lines are best-fit of the REDOR curves using equation S4 and the ^{29}Si-^{13}C dipolar coupling values given in Table 1. The vertical error bars correspond to the error analysis as given in section 3 above. The REDOR curves for each ^{29}Si sites are also shown separately in Figure S19.

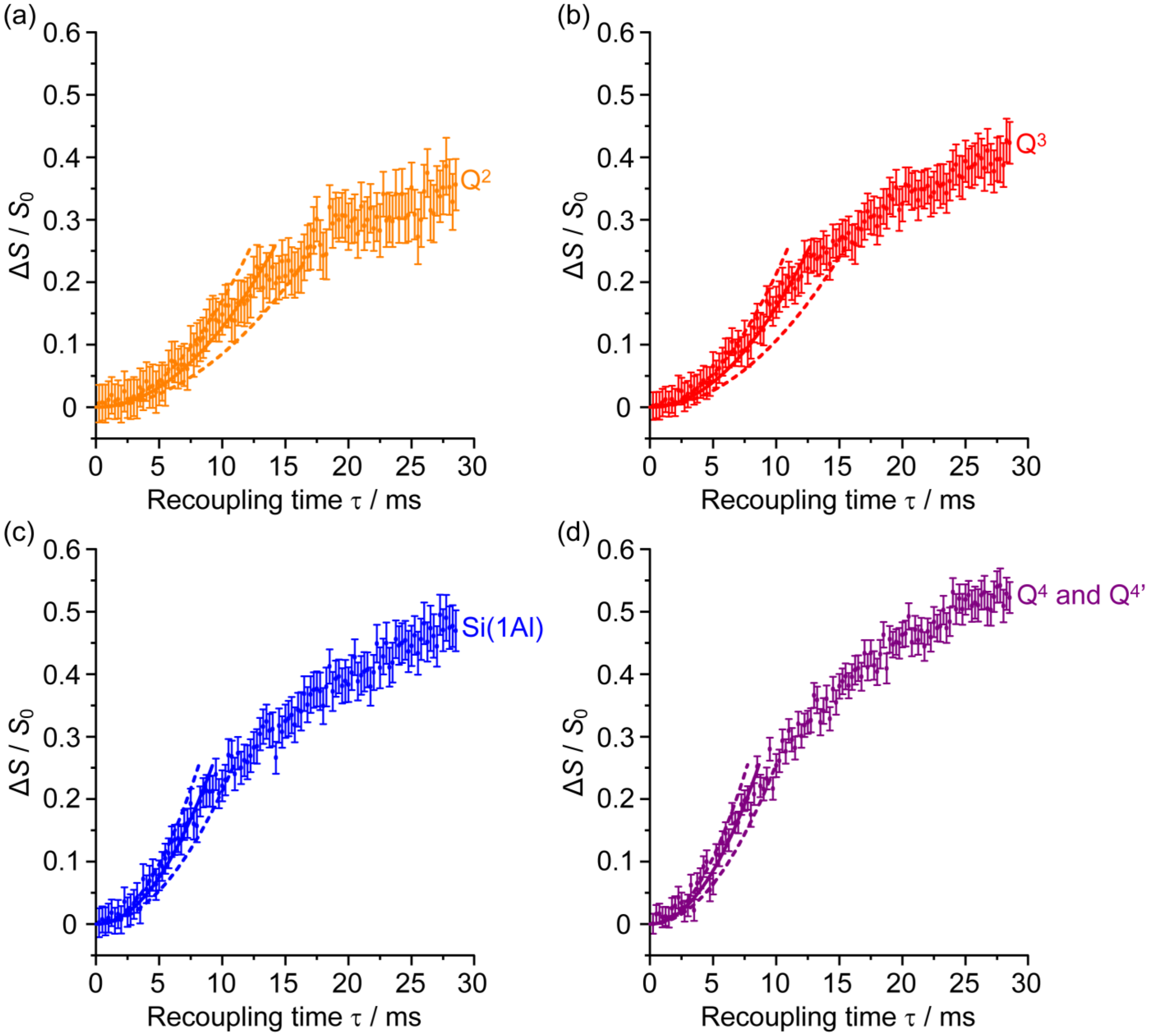

Figure S18. Individual ^{29}Si{^{13}C} REDOR curves for each ^{29}Si sites of activated M-β. The solid lines are best-fit of the REDOR curves (see Figure 7b) while the dashed lines correspond to the fit boundaries corresponding to the errors in the simulated ^{29}Si-^{13}C dipolar coupling values given in Table 1.

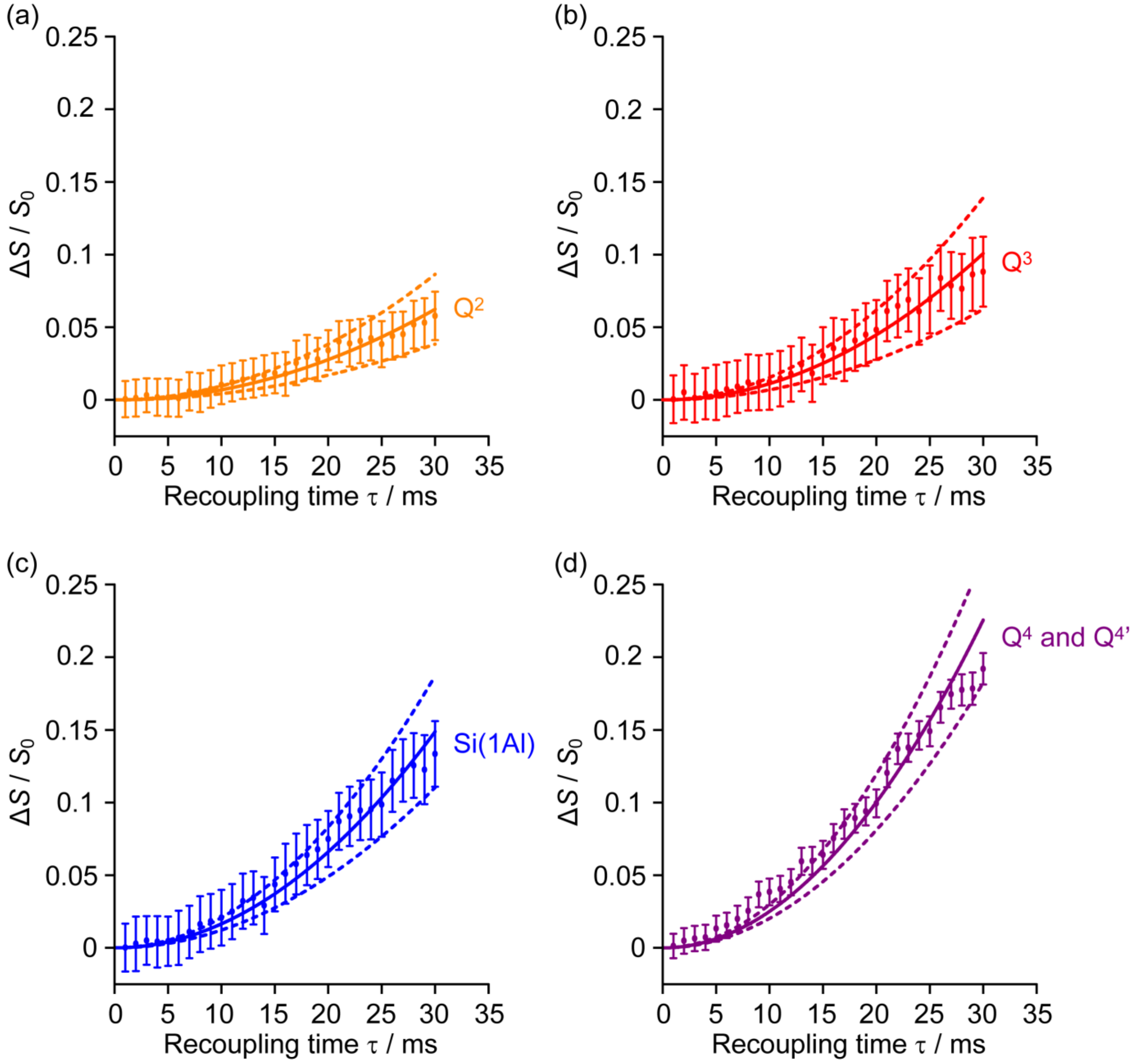

Figure S19. Individual $^{29}Si\{^{13}C\}$ REDOR curves for each ^{29}Si sites of activated MMM-β. The solid lines are best-fit of the REDOR curves (see Figure S17) while the dashed lines correspond to the fit boundaries corresponding to the errors in the simulated ^{29}Si-^{13}C dipolar coupling values given in Table 1.

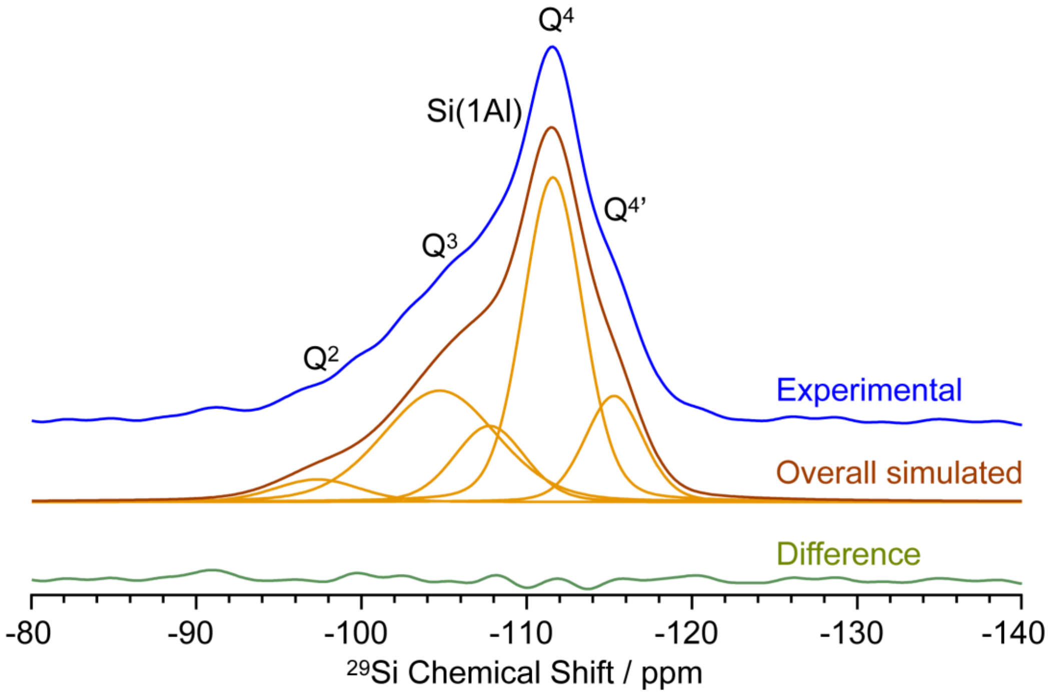

Figure S20. Quantitative room temperature ^{29}Si MAS NMR spectrum of H-type MMM-β at 9.4 T and a MAS rate of 10 kHz.

S6. References

1 L.-H. Chen, X.-Y. Li, G. Tian, Y. Li, H.-Y. Tan, G. Van Tendeloo, G.-S. Zhu, S.-L. Qiu, X.-Y. Yang and B.-L. Su, *ChemSusChem*, 2011, **4**, 1452–1456.

2 M. Zhang, S. Xu, J. Li, Y. Wei, Y. Gong, Y. Chu, A. Zheng, J. Wang, W. Zhang, X. Wu, F. Deng and Z. Liu, *J. Catal.*, 2016, **335**, 47–57.

3 B. M. Fung, A. K. Khitrin and K. Ermolaev, *J. Magn. Reson.*, 2000, **142**, 97–101.

4 G. Metz, X. Wu and S. O. Smith, *J. Magn. Reson., Ser. A*, 1994, **110**, 219–227.

5 A. Zagdoun, G. Casano, O. Ouari, M. Schwarzwälder, A. J. Rossini, F. Aussenac, M. Yulikov, G. Jeschke, C. Copéret, A. Lesage, P. Tordo and L. Emsley, *J. Am. Chem. Soc.*, 2013, **135**, 12790–12797.

6 A. Zagdoun, A. J. Rossini, D. Gajan, A. Bourdolle, O. Ouari, M. Rosay, W. E. Maas, P. Tordo, M. Lelli, L. Emsley, A. Lesage and C. Copéret, *Chem. Commun.*, 2012, **48**, 654–656.

7 K. R. Thurber and R. Tycko, *J. Magn. Reson.*, 2009, **196**, 84–87.

8 A. Lesage, M. Bardet and L. Emsley, *J. Am. Chem. Soc.*, 1999, **121**, 10987–10993.

9 N. M. Szeverenyi, M. J. Sullivan and G. E. Maciel, *J. Magn. Reson.*, 1982, **47**, 462–475.

10 K. Takegoshi, S. Nakamura and T. Terao, *Chem. Phys. Lett.*, 2001, **344**, 631–637.

11 C. R. Morcombe, V. Gaponenko, R. A. Byrd and K. W. Zilm, *J. Am. Chem. Soc.*, 2004, **126**, 7196–7197.

12 T. Gullion, *Concepts Magn. Reson.*, 1998, **10**, 277–289.

13 A. L. Webber, A. J. Pell, E. Barbet-Massin, M. J. Knight, I. Bertini, I. C. Felli, R. Pierattelli, L. Emsley, A. Lesage and G. Pintacuda, *ChemPhysChem*, 2012, **13**, 2405–2411.

14 D. Massiot, F. Fayon, M. Capron, I. King, S. Le Calvé, B. Alonso, J.-O. Durand, B. Bujoli, Z. Gan and G. Hoatson, *Magn. Reson. Chem.*, 2002, **40**, 70–76.

15 M. Rosay, L. Tometich, S. Pawsey, R. Bader, R. Schauwecker, M. Blank, P. M. Borchard, S. R. Cauffman, K. L. Felch, R. T. Weber, R. J. Temkin, R. G. Griffin and W. E. Maas, *Phys. Chem. Chem. Phys.*, 2010, **12**, 5850–5860.

16 F. M. Vigier, D. Shimon, V. Mugnaini, J. Veciana, A. Feintuch, M. Pons, S. Vega and D. Goldfarb, *Phys. Chem. Chem. Phys.*, 2014, **16**, 19218–19228.

17 N. J. Brownbill, B. B. Reiner Sebastian Sprick, S. Pawsey, F. Aussenac, A. J. Fielding, A. I. Cooper and F. Blanc, *Macromolecules*, 2018, **51**, 3088–3096.

18 A. J. Rossini, A. Zagdoun, M. Lelli, D. Gajan, F. Rascón, M. Rosay, W. E. Maas, C. Copéret, A. Lesage and L. Emsley, *Chem. Sci.*, 2012, **3**, 108–115.

19 A. J. Rossini, A. Zagdoun, F. Hegner, M. Schwarzwälder, D. Gajan, C. Copéret, A. Lesage

and L. Emsley, *J. Am. Chem. Soc.*, 2012, **134**, 16899–16908.

20 H. Takahashi, D. Lee, L. Dubois, M. Bardet, S. Hediger and G. De Paëpe, *Angew. Chem. Int. Ed.*, 2012, **51**, 11766–11769.

21 K. R. Thurber and R. Tycko, *J. Chem. Phys.*, 2014, **140**, 184201.

22 F. Mentink-Vigier, S. Paul, D. Lee, A. Feintuch, S. Hediger, S. Vega and G. De Paëpe, *Phys. Chem. Chem. Phys.*, 2015, **17**, 21824–21836.

23 S. R. Chaudhari, P. Berruyer, D. Gajan, C. Reiter, F. Engelke, D. L. Silverio, C. Copéret, M. Lelli, A. Lesage and L. Emsley, *Phys. Chem. Chem. Phys.*, 2016, **18**, 10616–10622.

24 F. Mentink-Vigier, G. Mathies, Y. Liu, A.-L. Barra, M. A. Caporini, D. Lee, S. Hediger, R. G. Griffin and G. De Paëpe, *Chem. Sci.*, 2017, **8**, 8150–8163.

25 S. R. Chaudhari, D. Wisser, A. C. Pinon, P. Berruyer, D. Gajan, P. Tordo, O. Ouari, C. Reiter, F. Engelke, C. Copéret, M. Lelli, A. Lesage and L. Emsley, *J. Am. Chem. Soc.*, 2017, **139**, 10609–10612.

26 N. J. Brownbill, D. Gajan, A. Lesage, L. Emsley and F. Blanc, *Chem. Commun.*, 2017, **53**, 2563–2566.

27 M. Bertmer and H. Eckert, *Solid State Nucl. Magn. Reson.*, 1999, **15**, 139–152.

28 M. Bjørgen, U. Olsbye, D. Petersen and S. Kolboe, *J. Catal.*, 2004, **221**, 1–10.

29 M. Bjørgen, S. Akyalcin, U. Olsbye, S. Benard, S. Kolboe and S. Svelle, *J. Catal.*, 2010, **275**, 170–180.

30 M. Bjørgen, U. Olsbye and S. Kolboe, *J. Catal.*, 2003, **215**, 30–44.

31 M. Bjørgen, U. Olsbye, S. Svelle and S. Kolboe, *Catal. Lett.*, 2004, **93**, 37–40.

32 J. Li, Y. Wei, J. Chen, P. Tian, X. Su, S. Xu, Y. Qi, Q. Wang, Y. Zhou, Y. He and Z. Liu, *J. Am. Chem. Soc.*, 2012, **134**, 836–839.

33 A. D. Chowdhury, K. Houben, G. T. Whiting, M. Mokhtar, A. M. Asiri, S. A. Al-Thabaiti, S. N. Basahel, M. Baldus and B. M. Weckhuysen, *Angew. Chem. Int. Ed.*, 2016, **55**, 15840–15845.

34 X. Wu, S. Xu, W. Zhang, J. Huang, J. Li, B. Yu, Y. Wei and Z. Liu, *Angew. Chem. Int. Ed.*, 2017, **56**, 9039–9043.

35 D. Lesthaeghe, J. Van der Mynsbrugge, M. Vandichel, M. Waroquier and V. Van Speybroeck, *ChemCatChem*, 2011, **3**, 208–212.
